# Stochastic dynamics of Type-I interferon responses

**DOI:** 10.1371/journal.pcbi.1010623

**Published:** 2022-10-21

**Authors:** Benjamin D. Maier, Luis U. Aguilera, Sven Sahle, Pascal Mutz, Priyata Kalra, Christopher Dächert, Ralf Bartenschlager, Marco Binder, Ursula Kummer

**Affiliations:** 1 Department of Modeling of Biological Processes, COS Heidelberg / Bioquant, Heidelberg University, Heidelberg, Germany; 2 Research Group “Dynamics of early viral infection and the innate antiviral response”, German Cancer Research Center (DKFZ), Heidelberg, Germany; 3 Division Virus-Associated Carcinogenesis, German Cancer Research Center (DKFZ), Heidelberg, Germany; 4 Department for Infectious Diseases, Molecular Virology, Medical Faculty, Heidelberg University, Heidelberg, Germany; University of Illinois at Urbana-Champaign, UNITED STATES

## Abstract

Interferon (IFN) activates the transcription of several hundred of IFN stimulated genes (ISGs) that constitute a highly effective antiviral defense program. Cell-to-cell variability in the induction of ISGs is well documented, but its source and effects are not completely understood. The molecular mechanisms behind this heterogeneity have been related to randomness in molecular events taking place during the JAK-STAT signaling pathway. Here, we study the sources of variability in the induction of the IFN-alpha response by using MxA and IFIT1 activation as read-out. To this end, we integrate time-resolved flow cytometry data and stochastic modeling of the JAK-STAT signaling pathway. The complexity of the IFN response was matched by fitting probability distributions to time-course flow cytometry snapshots. Both, experimental data and simulations confirmed that the MxA and IFIT1 induction circuits generate graded responses rather than all-or-none responses. Subsequently, we quantify the size of the intrinsic variability at different steps in the pathway. We found that stochastic effects are transiently strong during the ligand-receptor activation steps and the formation of the ISGF3 complex, but negligible for the final induction of the studied ISGs. We conclude that the JAK-STAT signaling pathway is a robust biological circuit that efficiently transmits information under stochastic environments.

## Author summary

We investigate the impact of intrinsic and extrinsic noise on the reliability of interferon signaling. Information must be transduced robustly despite existing biochemical variability and at the same time the system has to allow for cellular variability to tune it against changing environments. Getting insights into stochasticity in signaling networks is crucial to understand cellular dynamics and decision-making processes. To this end, we developed a detailed stochastic computational model based on single cell data. We are able to show that reliability is achieved despite high noise at the receptor level.

## Introduction

The interferon (IFN) system is the first line of innate immune defense. IFNs are polypeptides secreted by infected cells, inducing cell-intrinsic antimicrobial states that limit the spread of infectious agents. IFNs particularly act against viral pathogens in infected and neighboring cells. There are three distinct IFN families. The type-I IFN family comprises IFN-*β* and various subtypes of IFN-*α*. Most cell types produce IFN-*β* upon viral infection, whereas plasmacytoid dendritic cells are the predominant producers of IFN-*α* [1]. IFN*γ* is the only type-II IFN, and the type-III IFN family, the latest to be discovered, comprises IFNλ1–4. This study focuses on the widely studied type-I IFNs.

**Fig 1:**
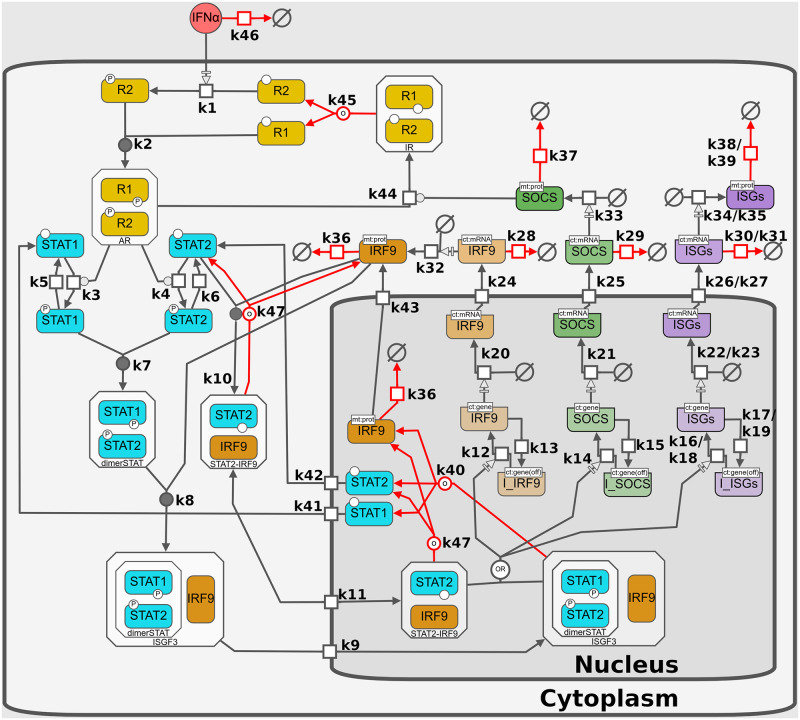
IFN activation of the JAK-STAT signaling pathway. Free IFN binds to the IFNAR subunits 1 and 2 to form an active complex. After IFNAR activation the signal is transduced inside the cytoplasm; here STAT1 and STAT2 are phosphorylated. Phosphorylated forms of STAT1 and STAT2 form a heterodimer (dimerSTAT). dimerSTAT interacts with IRF9 to form ISGF3 (a trimolecular complex). ISGF3 translocates to the nucleus. In the nucleus, ISGF3 binds to free transcription factor binding sites (ISRE), inducing transcriptional activity leading to production of more IRF9 as a positive feedback and SOCS as a negative feedback given by the SOCS1 degradation of active receptors. ISGF3 induces the expression of around 350 different ISGs, including MxA and IFIT1. Additionally, there is a constitutive formation of STAT2-IRF9 heterodimers, that stimulate the expression of interferon-induced genes (ISGs) without a signaling requirement (basal expression). The model consists of 42 species in two compartments and 62 kinetic reactions and is fully described in the methods section. In the figure, boxes represent the chemical species, empty symbols represent degradation processes, arrows represent the reactions described in Section B.1 in S1 Text, initial conditions and parameters are given in Tables 1, 2 and 3, respectively. The pathway diagram was created with the Newt Editor [26, 27] following the Systems Biology Graphical Notation (SBGN) [28].

The IFN system is tightly regulated, as aberrant or excessive IFN responses can be detrimental and contribute to the pathogenesis of autoimmune diseases [2]. To prevent over-activation, multiple mechanisms determine the efficiency and flexibility in IFN-mediated responses, by amplifying or suppressing IFN-dependent signaling responses. Positive regulation of signaling includes the triggering of the JAK-STAT signaling pathway by binding of extracellular type-I IFN to the IFN-*α* receptor (IFNAR). Upon ligation, the two receptor chains, IFNAR1 and IFNAR2, heterodimerize and activate the receptor-associated protein tyrosine kinases Janus kinase 1 (JAK1) and tyrosine kinase 2 (TYK2), which in turn phosphorylate the signal transducers and activators of transcription 1 (STAT1) and STAT2. Phosphorylated STAT1 and STAT2 heterodimerize and together with IFN-regulatory factor 9 (IRF9) assemble the ternary complex called IFN-stimulated gene factor 3 (ISGF3) that translocates into the nucleus. ISGF3 acts as a transcription factor that directly activates the transcription of a set of several hundred IFN-stimulated genes (ISGs) and thus induces a cellular antiviral state [3]. IRF9 also constitutes a crucial positive feedback of JAK-STAT signaling, as its initial concentration is limiting and subsequently increased by ISGF3 activity [4]. Reciprocally, negative feedback regulation of the IFN response has also been well established and includes the induction of negative regulators in the JAK-STAT pathway, such as suppressor of cytokine signaling 1 (SOCS1) [3]. In addition to the receptor-dependent activation of ISGs, there is also an IFN-independent basal expression caused by constitutive formation of STAT2–IRF9 that translocates to the nucleus and initiates the transcription of many ISGs [5]. After IFN treatment, STAT2-IRF9 complexes are largely replaced by tyrosine-phosphorylated STAT1–STAT2 heterodimers, which have a lower dissociation rate and a higher quantity [5]. A graphical description of the IFN-induced response is given in Fig 1.

The expression of MxA and IFIT1 (previously referred to as ISG56) are gold standards for surrogate markers representing IFN-*α*’s biological activity in various experimental and clinical settings [6, 7]. MxA is one of the most commonly studied ISGs. Its expression is highly induced by IFN and it exerts strong antiviral activity against Orthomyxoviruses, most prominently Influenza A virus, but also other families of negative-strand RNA viruses such as Rhabdoviridae or Paramyxoviridae [8–10]. IFIT1 on the other hand was one of the first ISGs to be discovered, is the most prominent member of the viral stress-inducible gene family and is strongly induced in response to type-I IFN as well as various viruses [11–13].

As in many signalling pathways with low participating species concentrations, stochasticity certainly plays a role in the information processing. Even though our knowledge about the role of stochasticity in biological communication processes increased, the interpretation of stochastic signal transducing processes remains a challenge. The expression of many ISGs is believed to follow a digitalized (bi-modal all-or-none) expression pattern [14–18], however several studies are indicating that there are also ISGs that follow a graded (unimodal) pattern [19, 20]. Moreover, the number and percentage of ISGs displaying a bimodal expression varies between species and cell types [21–25], which is believed to reflect functionally important differences [15]. Despite evidence of all-or-nothing responses for MxA and IFIT1 gene induction [14, 15], the stochastic behavior of these genes cannot be fully explained yet. For instance, IFIT1 mRNA was shown to be bimodal in early time points before transitioning to a uniform response in Shalek et. al (2013) [15]. In order to answer this question and to better understand pathway information processing, the behaviour of these IFN activity markers has to be further verified. In the case of the IFN stimulated genes, neither the mechanisms nor the function of intrinsic variability in discrete reactions along the IFN activated pathway are well understood.

Previously, the dynamics of the JAK-STAT signaling pathway have been deterministically modeled and validated with time course data describing average dynamics in cell populations [4, 29–31]. Many questions could be answered with these studies. However, looking at the experimental data from single-cell measurements, it is obvious that stochastic influences play a major role. These data show distributions of behaviour rather than deterministic phenotypes. Obviously, the above efforts lack an exhaustive and systematic analysis of the influence of such stochastic fluctuations in the molecular reactions taking place along the pathway. The term “*biochemical noise*” is used to describe the inherently discrete and random interactions of molecules in the cellular environment [32]. Total biochemical noise is conventionally divided into two components: intrinsic and extrinsic noise. Intrinsic noise results from the probabilistic nature of molecular processes. Extrinsic noise results from (cell-to-cell) variations in the concentrations of cellular biomolecules such as ribosomes, enzymes, metabolites, overall proteins and nucleic acids [33]. Extrinsic and intrinsic noise have been proven to critically affect cellular decision-making processes [34]. Plenty of research has revealed genetic circuits that are subject to noise at the level of their components. In contrast, little is known about the effect of noise in cellular signaling pathways. However, previously, it has been shown that the information transduced in cellular signaling pathways is affected by noise [35]. Noise in cellular signaling pathways is conceptually different from the noise resulting from stochastic fluctuations of low-abundance genetic regulators. In cellular signaling pathways, noise can consist of low levels of enzyme activity through random transient interactions of receptors, binding of ligands to receptors in nonfunctional complexes, and background interactions along the pathway [36]. Noise plays an important role in IFN responses. It has been observed that within populations of nearly identical cells, not all cells that are infected by a virus or stimulated by IFN express ISGs [14, 37–41]. In all these cases the suggested mechanisms behind this heterogeneity has been related to stochastic events along the JAK-STAT signaling pathway.

Live-cell imaging data is indicating a multi-layered stochasticity (viral replication, IFN induction and IFN response), whereby heterogeneous IFN induction is largely determined by the translocation time of the key transcription factors and cell-intrinsic noise [14]. This result is challenged however by a recent integrated ChIP-seq and transcriptome analysis, which found that the molecular switch from an unstimulated homeostasis to a strong IFN-induced receptor-dependent response is caused by a rapid exchange of unphosphorylated transcription factors for the phosphorylated key transcription factors [5]. Furthermore, their results indicate that unstimulated and activated state are not only differing by intensity as previously assumed but also mechanistically as different signaling cascades are used, which has not been taken into account in earlier mathematical models and might be crucial to understand the above described (cell-intrinsic) noise. Hence, this study attempts to achieve greater detail than previous stochastic models, using more readily available flow cytometry data and novel literature findings.

Little is known about the sources and principles behind basal activity and expression in the JAK-STAT signaling pathway. Studies suggest that constitutive expression varies greatly between different cell types and occurs at different levels (receptor, signal transduction, gene expression) [42]. Basal STAT phosphorylation in diseased cells and tissues has been demonstrated by several groups [43–46]. Recently, it was shown that the basal expression of many interferon-induced genes (ISGs) is stimulated by STAT2–IRF9 complexes which form constitutively in the cytoplasm, shuttle between nucleus and cytoplasm and bind to the promoter region without a signaling requirement [5]. To which extent basal expression fluctuates within a population of genetically identical cells is, however, unknown so far.

Here, we aim to determine whether and how biochemical noise affects the information transduced in the JAK-STAT signaling pathway and investigate the transition between basal and activated state. To this end, we studied the stochastic responses of MxA and IFIT1 expression in Huh7.5 cells stimulated with IFN-*α*. Using fluorescent reporters under the control of the authentic promoter/enhancer region of IFIT1 and MxA we collected data displaying the differences between expressing and non-expressing cells for the marker genes in a time-course experiment. We hypothesize that the JAK-STAT signaling pathway efficiently transmits information under stochastic environments. To test our working hypothesis, we developed a detailed mathematical model using the obtained time-resolved flow cytometry data to describe the elements in the JAK-STAT signaling pathway at single-cell resolution. This model allowed us to systematically test the influence of intrinsic and extrinsic noise in the IFN response. We determined that the effects of intrinsic noise are particularly strong at the receptor level, during the formation of the transcription factor ISGF3 and during the transcription of the ISGs. We concluded that the JAK-STAT signaling pathway is a robust system that can filter extrinsic fluctuations.

## Materials and methods

### Experimental set up

The data described the expression of MxA and IFIT1 after IFN-*α* stimulation in a population of Huh7.5 cells. The experiments were done in the following way: Cells harboring a BAC (Bacterial Artificial Chromosome) containing the studied ISGs (mxa and ifit1) fused with the destabilized enhanced green fluorescent protein (deGFP) were seeded into a 6-well plate with 1.5*x*10^5^ cells per well. Next day, cells were treated in a time-dependent manner with IFN-*α* (8, 12, 16, 20 and 32 h) and harvested at the same time point. Cells were fixed with 2% paraformaldehyde to stop further protein expression for 10 min, washed three times with PBS and applied for flow cytometry (BD Accuri C6). Measuring GFP fluorescence intensity served as marker for ISG induction. Data was evaluated with the FlowJo v10 software package. Human IFN-*α* (Alpha 2a) was obtained from Pbl Assay Science with a reported specific activity of 3.91*x*10^8^ units/mg.

### Mathematical model of the JAK-STAT signaling pathway upon IFN-*α* stimulation

The developed model consists of 42 species and 62 reactions (reactions m1 to m62). To name the variables in the model we used the following conventions: 1) variables referring to mRNA are denoted by *m* prefix. 2) Variables in phosphorylated use *p* as prefix. 3) Gene promoters are represented by the gene’s name in lowercase. 4) R1, R2, IR, AR and RC, represent the IFN receptor subunits, inactive, active and complex forms, respectively. 5) The compartment is superscripted to the species if the species exist in multiple compartments. A graphical representation of the interaction between variables in the model is given in Fig 1 and all reactions are listed in the Section B.1 in S1 Text. The model can be accessed via Biomodels: https://www.ebi.ac.uk/biomodels-main/ under reference: MODEL2210070001.

### Mapping the system dynamics to experimental deterministic data

The measurements of time course data represent the average dynamics of proteins in a population of Huh7.5 cells [4]. Experimental data measurements were made comparable with the corresponding observable chemical species *S*^*O*^ in the model by a function *h* that maps state *S*^*O*^(*t*_*i*_) to observation *S*^†^(*t*_*i*_) as follows:

$$\begin{array}{c} S^{†}\left(t_{i}\right)=h\left(S^{O}\left(t_{i}\right)\right), \end{array}$$ (1)

at each measurement time point *t*_*i*_, *i* = 1, …, *n*; where *n* is the total number of observable time points.

Subsequently, average data measurements were made comparable with the corresponding observable chemical species in the model, considering a specific set of parameter values *θ* = {*θ*_1_, …, *θ*_*d*_}, by the use of the following objective function:

$$\begin{array}{c} F_{D}\left(\theta ,S^{O}\right)=\sum_{i=1}^{m}\sum_{j=1}^{n}{\left(S_{ij}^{†}-S_{ij}^{O}\left(\theta \right)\right)}^{2}. \end{array}$$ (2)

Eq (2) compares the time courses for $S_{ij}^{†}$ and $S_{ij}^{O}$, and the difference between simulated and experimental data. A full description of the functions and scaling parameters used to map systems dynamics to experimental data is given in the Section C.1 in S1 Text.

### Fitting the stochastic system to flow cytometry data

The process to fit the stochastic system to flow cytometry data was developed based on [47] and [48]. In short, empirical cumulative density functions (ECDF) were built for experimental data and simulations results. First, having *nm* repetitions of single-cell experimental data from flow cytometry measurements at *I* time points *t*_*i*_, i = 1, …, *I*, that is ***m***(*t*_*i*_) = {*m*_1_(*t*_*i*_), …, *m*_*nm*_(*t*_*i*_)} ECDFs for the experimental data ${\hat{F}}_{e}\left(m\left(t_{i}\right)\right)$ were built. In a similar way, considering a specific set of parameter values *θ* = {*θ*_1_, …, *θ*_*d*_}, we performed *ns* repetitions of the stochastic simulations ***s***(*t*_*i*_) = {*s*_1_(*t*_*i*_), …, *s*_*ns*_(*t*_*i*_)}. The total of those stochastic simulations were used to build the ECDF for each *t*_*i*_ that is ${\hat{F}}_{s}\left(s\left(t_{i}\right),\theta \right)$. To calculate the distance between ${\hat{F}}_{e}$ and ${\hat{F}}_{s}$ we used the Kolmogorov distance (*D*_*KS*_), that is the absolute value of the difference between the two distributions. For ${\hat{F}}_{e}$ and ${\hat{F}}_{s}$ their Kolmogorov distance is:

$$\begin{array}{c} D_{KS}=\underset{x}{\text{max}}\left|{\hat{F}}_{e}-{\hat{F}}_{s}\right| \end{array}$$ (3)

### Parameter estimation strategy

Parameter searches consisted on optimization routines based on genetic algorithms implemented in R [49–51]. The proposed method improves its performance by selecting parameters values after comparing the similitude between the first statistical moment of the system and the first statistical moment in the experimental data distribution. By this pre-selection of parameter values most of the original parameters are rejected and the algorithms focus on the finding of parameters that reproduce the observed distribution dynamics [48]. The full strategy for parameter estimation is given in the Section C in S1 Text.

### Numerical methods

Raw experimental data was analyzed using the function *FCS data reader* coded in Matlab [52]. Statistical moments were computed using R 4.1. The parameter estimation was performed using the genetic algorithm from the package “GA” coded in R [50, 51] and the COPASI R Connector [49]. Intrinsic noise was introduced by solving the model under stochastic dynamics using the adaptive explicit-implicit tau-leaping method with automatic tau selection [53] coded in COPASI 4.30 [54]. Extrinsic noise was simulated following the method suggested by Shahrezaei et al., [55] that proposes a modification of the standard SSA introducing extrinsic variability in the model parameters by random sampling a distribution. For our model, we focus our attention on understanding the effect of extrinsic variations in the initial conditions. For this, we randomly sampled a Normal distribution $N(\mu ,\;\sigma^{2})$ with values for *μ* given in Table 1 and one of three values of *σ*: 0, 0.3, 0.6. In a final step, a parameter-robustness analysis was carried out to determine to what extent the evaluated system functionality is preserved under considered perturbations. Parameters were altered between half and double their original values individually and after subsequent stochastic simulations, the effect on the ISG induction was quantified using the KS-distance.

**Table 1:** System’s initial conditions (IC).

Variable	IC (Molecules/Cell)	References
^†^IFN	[5 × 10^3^, 1 × 10^4^]	Fitted
R2	1000	[56–59]
RC	0	TS
R1	1000	TS
AR	0	TS
STAT1_c_	850667	[4, 30, 60, 61]
STAT1_n_	0	TS
^‡^STAT2_c_	9325	[4, 60, 62]
^‡^STAT2_n_	150	[4, 60, 62]
SOCS	0	[63]
dimerSTAT	0	[64]
IRF9_c_	10	[4]
^‡^IRF9_n_	60	Fitted
ISGF3_c_	0	TS
ISGF3_n_	0	TS
^‡^STAT2-IRF9_c_	200	Fitted
^‡^STAT2-IRF9_n_	325	Fitted
pSTAT1	0	[64]
pSTAT2	0	[64]
IR	0	TS
^‡^mIRF9_c_	14	Fitted
mIRF9_n_	0	TS
mSOCS_c_	0 (avg. 0.08)	TS
mSOCS_n_	0	TS
mMxA_c_	0 (avg. 0.2)	TS
mMxA_n_	0	TS
mIFIT1_c_	0 (avg. 0.2)	TS
mIFIT1_n_	0	TS
^⋆^ *MxA*	1582	Fitted
^⋆^*IFIT*1	705	Fitted
irf9	0	TS
socs	0	TS
ifit1	0	TS
mxa	0	TS
irf9*	0.62	TS
socs*	0.02	TS
mxa*	0.06	TS
ifit1*	0.11	TS
I_irf9_	0.38	TS
I_socs_	0.98	TS
I_mxa_	0.94	TS
I_ifit1_	0.89	TS

^†^ Values for the initial IFN concentration were estimated by fitting the model to the experimental immunoblotting data. Obtaining that 500 U of IFN correspond to 10,000 IFN molecules and 250 U of IFN correspond to 5,000 molecules.

^‡^ System’s initial conditions for IRF9_*n*_, mIRF9_*c*_ and STAT2-IRF9 complexes as well as localisation distributions of STAT2 were estimated such that the model is in a steady state in absence of interferon under consideration of experimental measurements, information about retention signals and localisation distributions from other groups [4, 5].

^⋆^ Values for the initial ISG particle numbers were estimated by fitting the model to the experimental FACS data.

This study (TS) represent values assumed for the simulations: All species which are believed to be inactive in absence of stimulation and are not driving the basal expression were zeroed, while the initial conditions of two-state promoter scheme were set to the unstimulated steady state (just basal expression). The molecule number of R1 was assumed to match the known literature value of R2.

## Results

### Heterogeneity in IFN responses

**Fig 2:**
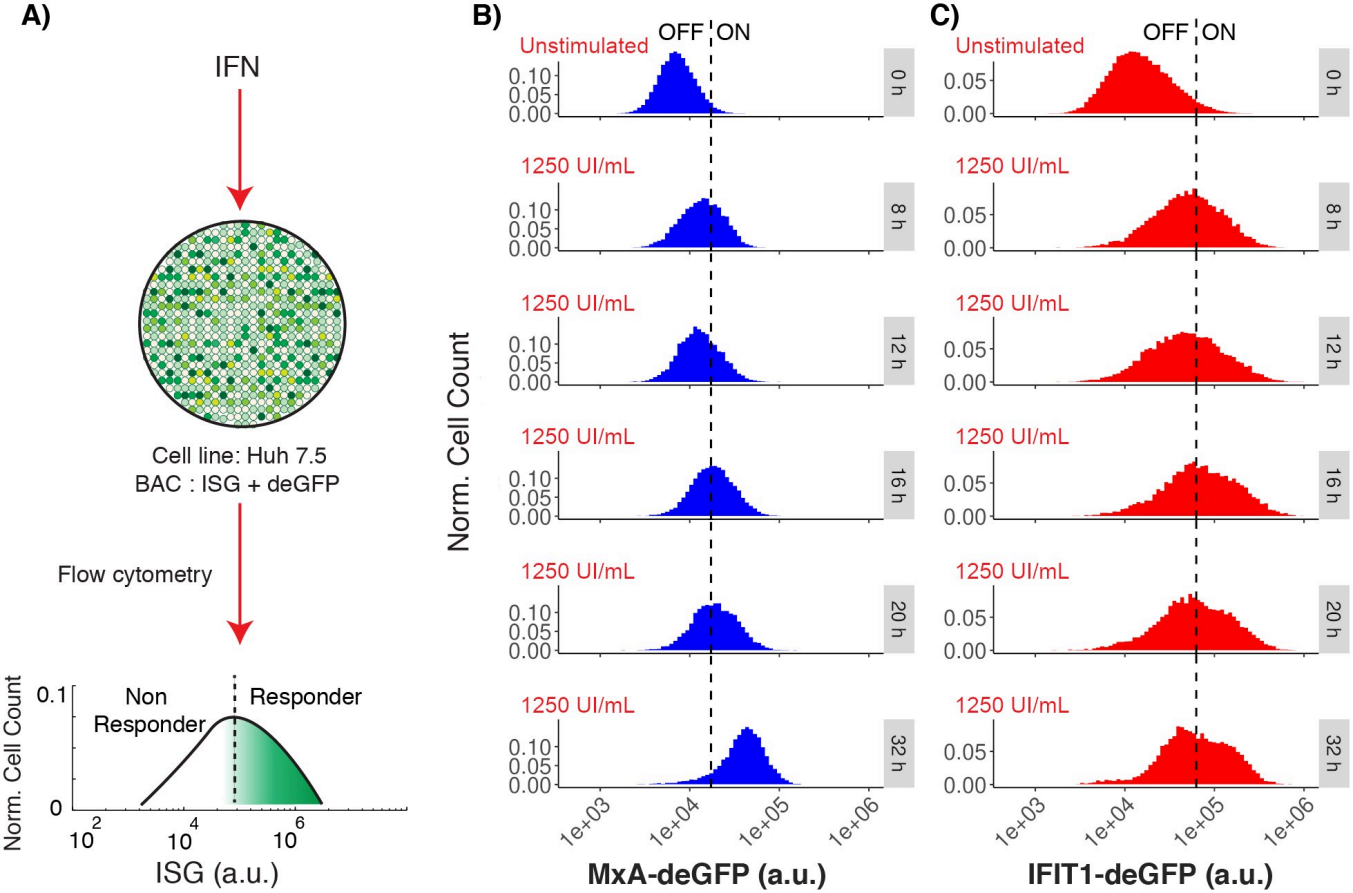
ISG induction after IFN stimulation. A: Experimental set-up showing the graded expression of MxA and IFIT1 after IFN-*α* stimulation in a population of Huh7.5 cells. A threshold is defined to differentiate responder vs. non-responder cells in the population. B: Distributions represent the flow cytometry measurements of MxA expression at different time points after IFN stimulation (blue distribution). C: Distributions represent the flow cytometry measurements of IFIT1 expression at different time points after IFN stimulation (red distribution). In figures B and C, the vertical dashed lines represents a threshold value (defined as the mean plus two standard deviations) calculated form the unstimulated populations. This threshold value is used to differentiate responser vs non-responder cells in a cell population with a graded response. In the observed treatments, the mean fluorescence level shifts from 8*x*10^3^ a.u. (arbitrary units of fluorescence) to 4.5*x*10^4^ a.u. for MxA, and from 2*x*10^4^ to 9*x*10^4^ a.u. for IFIT1.

To analyze the dynamics of the IFN signaling pathway, we employed Huh7.5 cells stably transfected with bacterial artificial chromosomes (BACs) containing MxA or IFIT1, respectively, tagged with a destabilized enhanced green fluorescent protein (deGFP) in their genuine genomic context, including up- and downstream regulatory sequences as we described previously [38, 40]. This reporter system allowed for fluorescence activated flow cytometry (FACS) based quantification of the authentic induction of the respective ISG expression upon IFN stimulation. We implemented a work-flow to measure fluorescence from the reporter genes at a single-cell level by FACS and their distribution in the cell population, thereby capturing cell-to-cell variability of the IFN response (Fig 2A). Our results indicated that, both, MxA and IFIT1 sustained graded responses, and heterogeneity in the IFN response was observed by the deformation and shifting of an unimodal distribution. In this unimodal distribution, responder cells are in the upper percentile and the center of the curve, and the non-responder cells are in the lowest percentile. We observed that IFN stimulation shifted the whole distribution to higher fluorescence intensities, i.e. ISG concentrations (Fig 2B and 2C). This contrasts somewhat with previous publications indicating an all-or-none switch-like response with two discernible subpopulations of responder and non-responder cells, based on IRF7 that was used as the reporting ISG [14]. However, apart from the ISG, also the cell system and species (mouse) was different than in our study, suggesting a possible cell-type or species specific response behavior.

We further tested in what way different concentrations of IFN affect the obtained graded response by comparing different doses of IFN (0, 10, 50, 250, 1250 UI/mL) and different time points (0, 8, 12, 16, 20 and 32 hours). We observed a dose-dependent shift of the population to higher signal intensities starting from 16 h post stimulation for MxA (Fig A in S1 Text) and from 12 h for IFIT1 (Fig B in S1 Text), but the graded responses were always maintained at different doses of IFN stimulation and for different time points. This may exclude the possibility of temporal monostable dynamics and then an all-or-none switch response. By plotting the mean fluorescence intensities (MFI) we analyzed time dependence of MxA and IFIT1 responses (Fig C in S1 Text). Our results indicated that IFIT1 reached its maximum response after 16 hours of IFN stimulation, whereas the maximum response for MxA was obtained at 32 hours of treatment. In summary, our results confirmed that MxA and IFIT1 only support graded responses, that those responses- as expected- depended on the dose of IFN used to stimulate the cells, and that MxA and IFIT1 exhibited distinct temporal induction dynamics.

### Stochastic modeling of the JAK-STAT signaling pathway and parameter estimation

In order to develop a deeper understanding of the JAK-STAT signaling pathway we devised a new stochastic model. The developed mathematical model has single-cell resolution comprising two compartments, known key components, feedback responses, constitutive regulatory mechanisms and a basal expression. The model architecture is based on previous models that consider the well accepted topology of the pathway [4, 29–31]. Contrary to these previous models and a more recently published model [65], we constructed a stochastic model and implemented a detailed model of the basal expression. Additionally, the receptor-independent activation of interferon stimulated genes via STAT2-IRF9 heterodimers was included as recent studies identified them as key players for basal expression and rapid activation of the signaling pathway [5]. The developed model consists of 42 species and 62 kinetic reactions, the complete model is given in Fig 1. All enzymatic reactions are assumed to be mass action kinetics (Table A in S1 Text). Processes of gene induction were modeled following a two-state promoter scheme [66]. To solve the model, we used the Adaptive Stochastic Simulations Algorithm (SSA) with *τ*-leap approximation [53, 67], which was shown to lead to the same results as the standard SSA algorithm. This was also confirmed by us, as can be seen in Fig D in S1 Text. Extrinsic variability in protein concentration was introduced in the initial conditions by random sampling from a normal distribution following the scheme introduced by Shahrezaei et al. [55].

**Fig 3:**
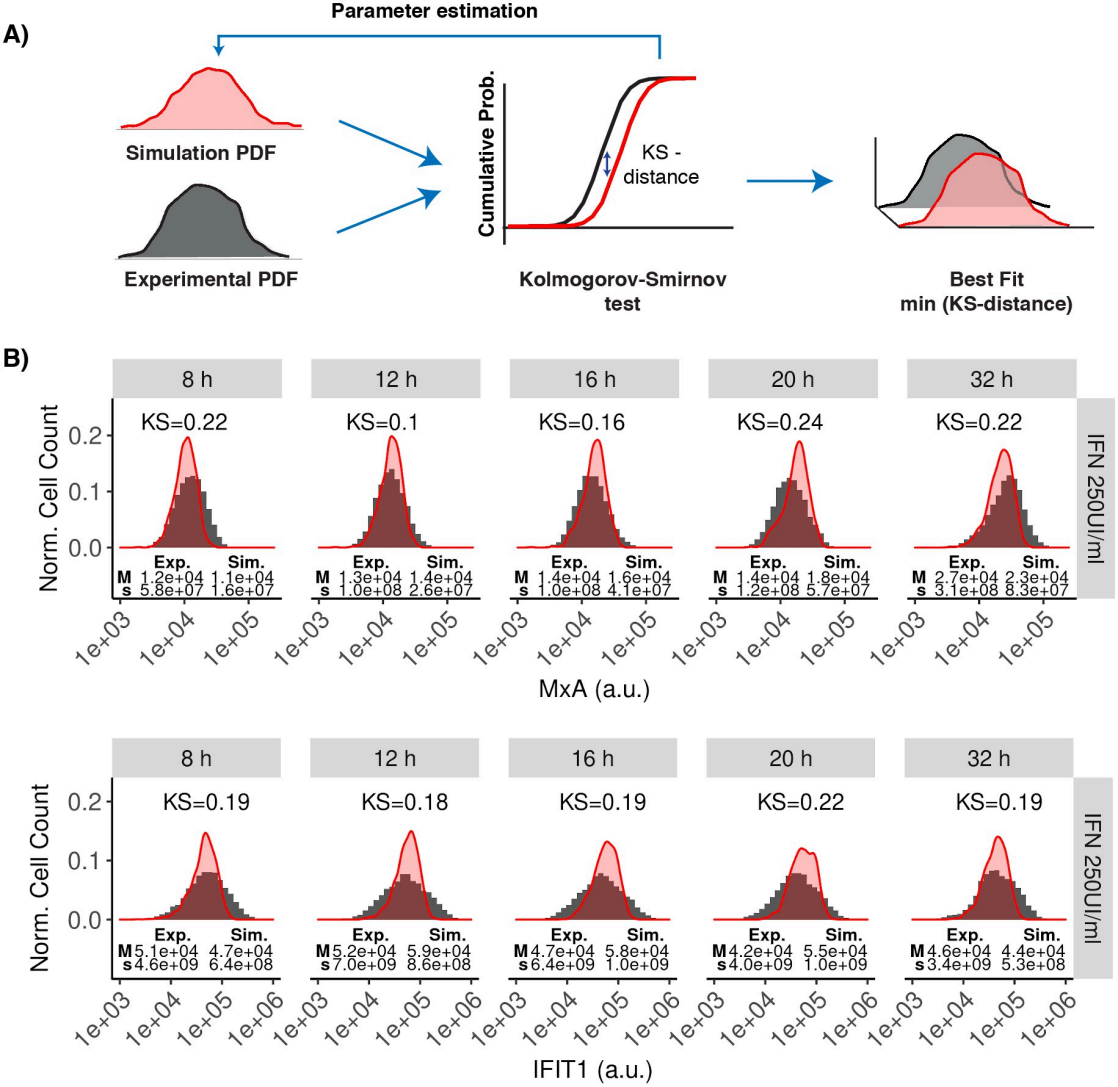
Fitting single-cell data to the stochastic model. A: The parameter estimation strategy consist of optimization routines based on genetic algorithms. The proposed methods measure the similitude between experimental and simulated distributions using KS-distance. The best parameters are obtained by minimizing the KS-distance. The full strategy for parameter estimation is given in the Section C in S1 Text. B: Experimental time-dependent distributions were computed from the flow cytometry datasets (filled histograms). Simulated time-dependent distributions were computed by solving our model under stochastic dynamics and repeating the simulations 1,000 times (red density plots). In the plots, the y-axis represents the normalized cell count and the x-axis represents the fluorescence quantity (arbitrary units, a.u.) associated with the expression of the MxA and IFIT1 proteins at various time points after stimulating Huh7.5 cells with 250 UI/mL IFN (5,000 IFN molecules). For each distribution, the median (M) and variance (s) is given. The initial conditions are given in Table 1, fitted parameter values are given in Table 2 and compartment sizes are given in Table 3. See Figs H and I in S1 Text for fits using different IFN doses.

**Fig 4:**
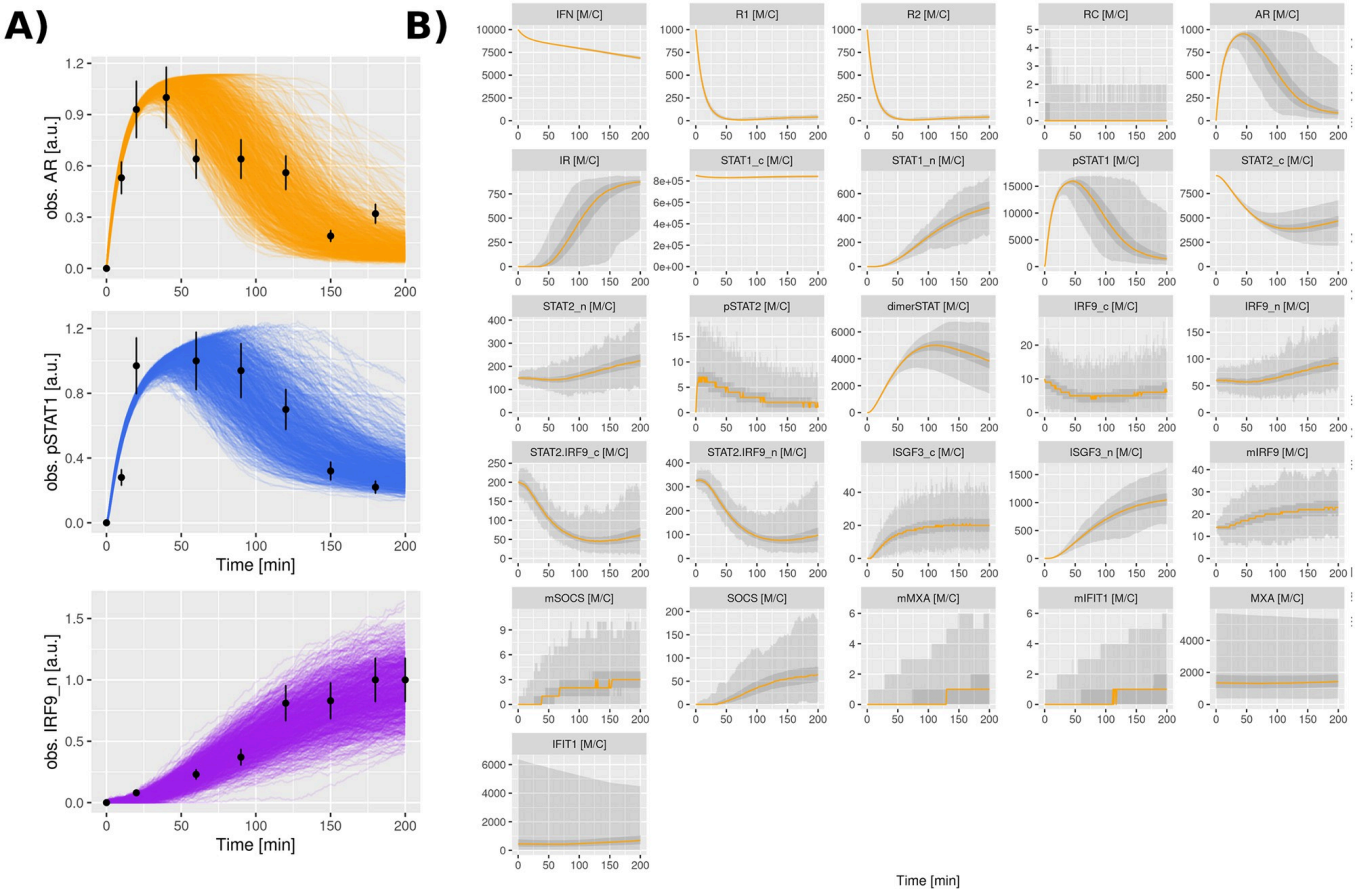
Model temporal dynamics and cell population data. A: Cell population data describing the temporal dynamics of phosphorylated JAK1, pSTAT1 and nuclear IRF9. Experimental data describe quantitative immunoblotting measurements in Huh7.5 cells after stimulation with 500 UI/mL of IFN-*α* at different time points for a total time of 180 min [4]. Measurement error of 18% is represented in the figures as error bars. Model dynamics were obtained by repeating the stochastic simulation 1,000 times, each trajectory representing a single cell. The procedure for mapping the model variables and experimental data is given in Section C.1 in S1 Text. B: Time courses data describing the temporal dynamics of all species involved in the JAK-STAT signaling pathway. The plots represent the repetitions of the stochastic model. The y-axis has units of Molecules per Cell (M/C). Orange lines represent the median, while light gray display the range of values and dark gray ribbons contain 50% of the values. The ribbons may not be visible if there is almost no variation (e.g. IFN) or if there is no particle present for the majority of trajectories (e.g. mIRF9_*n*_).

To identify the model parameters, we made use of our recently developed algorithm that fits single-cell data to stochastic models [48]. The method uses a comparison based on Kolmogorov-Smirnov (KS)-distances of the cumulative density functions that are computed based on Monte Carlo simulations and the experimental data. Multiple parameter values are iteratively evaluated using genetic algorithms (Fig 3A). The complete parameter estimation method is described in the Section C in S1 Text. To increase certainty in our analysis we performed a bibliographic search to narrow down parameter space with already determined parameter values (Table 2). The parametrized model was capable to reproduce the observed IFN response for the full probability distributions for MxA and IFIT1 at five different time points and different doses (Fig 3B and Fig H in S1 Text). Please note that at higher doses the variance is slightly underestimated which leads to an overestimation of the amplitude of the distribution, since total numbers have to be the same. The fit could possibly be further improved by including important elements whose implications in the JAK-STAT signaling pathway have only recently been described and which were not considered in this model (for example basal expression of phosphorylated forms of STAT proteins [64], receptor recycling and endocytic regulation [68], and more elements involved in the negative feedback [3]). Including those additional elements in our model is possible in principle. Nevertheless, this would increase the number of free parameters in the system, probably causing overfitting problems as described in the Section D in S1 Text. The overall good agreement indicated that the model can capture the heterogeneity in the IFN response. We also tested our model for resolving the dynamics of the pathway at different levels of the signaling cascade. For that purpose, we exploited previously published population-level data for the activation of the key signaling components JAK1, STAT1 and IRF9, all measured in Huh7.5 cells stimulated with IFN-*α* [4]. Notably, the average results of our stochastic model largely matched the temporal response of those signaling components (Fig 4A). Hence we conclude that our model (using a single parameter set) based on two different experimental data sources (flow cytometry data and immunoblotting measurements) can faithfully reproduce the system’s dynamics.

**Table 2:** Description of the parameter values.

Parameter	Nominal	Units	References
k_1_	7.5 × 10^−6^	1/(Molecules⋅min)	Fitted
k_2_	1.32	1/(Molecules⋅min)	Fitted
k_3_	1.75 × 10^−5^	1/(Molecules⋅min)	Fitted
k_4_	1.34 × 10^−5^	1/(Molecules⋅min)	Fitted
k_5_	1.0	1/min	Fitted
k_6_	2.26	1/min	Fitted
k_7_	9.88 × 10^−4^	1/(Molecules⋅min)	Fitted
k_8_	7.59 × 10^−4^	1/(Molecules⋅min)	Fitted
k_9_	8.7 × 10^−1^	1/min	Fitted
k_10_	2.63 × 10^−4^	1/(Molecules⋅min)	Fitted
k_11.1_	2.2 × 10^−1^	1/min	Fitted
k_11.2_	2.5 × 10^−1^	1/min	Fitted
^⋆^k_12_	1.25 × 10^−3^	1/(Molecules⋅min)	Fitted
^⋆^k_13.1_	2.25 × 10^−3^	1/min	Fitted
^⋆^k_13.2_	2.0	1/min	Fitted
^⋆^k_14_	9.97 × 10^−4^	1/(Molecules⋅min)	Fitted
^⋆^k_15.1_	1.49	1/min	Fitted
^⋆^k_15.2_	107.5	1/min	Fitted
^⋆^k_16_	6.1 × 10^−4^	1/(Molecules⋅min)	Fitted
^⋆^k_17.1_	1.95	1/min	Fitted
^⋆^k_17.2_	22.8	1/min	Fitted
^⋆^k_18_	7.0 × 10^−4^	1/(Molecules⋅min)	Fitted
^⋆^k_19.1_	8.71 × 10^−1^	1/min	Fitted
*k* ^⋆^k_19.2_	10.6	1/min	Fitted
^‡^k_20_	3.28 × 10^−1^	1/min	[69]
^‡^k_21_	1.47 × 10^−1^	1/min	[69]
^‡^k_22_	1.34 × 10^−2^	1/min	[69]
^‡^k_23_	1.4 × 10^−2^	1/min	[69]
k_24_	8.76 × 10^−1^	1/min	Fitted
k_25_	4.89	1/min	Fitted
k_26_	1.38	1/min	Fitted
k_27_	1.04 × 10^−1^	1/min	Fitted
k_28_	1.39 × 10^−2^	1/min	[70]
k_29_	4.36 × 10^−2^	1/min	[70]
k_30_	2.38 × 10^−3^	1/min	[70]
k_31_	7.37 × 10^−3^	1/min	[70]
^†^k_32_	8.64 × 10^−1^	1/min	[71]
^†^k_33_	6.26 × 10^−1^	1/min	[71]
^†^k_34_	1.52	1/min	[71]
^†^k_35_	4.02	1/min	[71]
k_36_	1.73 × 10^−1^	1/min	Fitted
k_37_	2.5 × 10^−2^	1/min	[72]
k_38_	3.51 × 10^−4^	1/min	[4]
k_39_	1.95 × 10^−3^	1/min	[73, 74]
k_40_	1.85 × 10^−2^	1/min	[72]
k_41_	1.18 × 10^−2^	1/min	Fitted
k_42_	5.09 × 10^−3^	1/min	Fitted
k_43_	1.43 × 10^−2^	1/min	Fitted
k_44_	5.05 × 10^−4^	1/(Molecules⋅min)	Fitted
k_45_	2.94 × 10^−3^	1/min	Fitted
k_46_	1.2 × 10^−3^	1/min	[56, 75]
k_47_	4.77 × 10^−2^	1/min	Fitted

^⋆^ Transcription factor dissociation rates for STAT2-IRF9 were assumed to be substantially higher than for ISGF3, while the association rates were assumed to be identical [5].

^†^ Translations rates were assumed to be close to reported averages times ≈ 16 Proteins/mRNA/min [71].

^‡^ Transcription rates were assumed to be close to reported averages times to produce a mRNA ≈ 20 min [69].

IFNR dissociation constant of 10^−9^,10^−11^ [57]. RNA half-life was assumed close to the average value reported by [70].

**Table 3:** Compartment sizes.

Compartment	Volume [%]	References
Cytoplasm	86.5	[76–80]
Nucleus	13.5	[77–79, 81]

Typically, human hepatocytes have a volume about 3500 *μ*m^3^ [82] and display a low nucleus-to-cytoplasm ratio (<15%) [77], whereas the presence of high N:C ratios indicate malignancy and is therefore commonly found across hepatocellular carcinoma cell lines. Literature values of nuclear-to-cytoplasmatic ratio (N:C ratio) for Huh7/Huh7.5 cells ranging between 10% and 45%. For the majority of measured Huh7 cells, the nuclear volume was quantified to be between 400–750 *μ*m^3^ [76], while the total cellular volume can be estimated to be between 1500 *μ*m^3^ [76] and roughly 4000 *μ*m^3^ [80]. Huh7.5 cells were assumed to be spherical in order to convert cell diameters in cell volume. Given the reported wide distributions of compartment sizes and the uncertainty in literature, we decided to proceed with the well accepted N:C ratio for healthy hepatocytes [78] and repeated our simulations with a nucleus double the initial size (27%). We concluded that the overall system dynamics in absence and under the influence of extrinsic are consistent to our findings even when doubling the nucleus size despite new steady state conditions (Fig O in S1 Text).

### Effect of noise in the ISG induction after IFN stimulation

Intrinsic noise is caused by the stochasticity of the involved processes meaning that there is variability even if the same initial conditions are used whereas extrinsic noise originates in the cell-to-cell variability of protein abundancies etc. In order to compare and analyse these two effects we computed repeated time-series for both scenarios.

#### Intrinsic noise in the JAK-STAT signaling pathway

**Fig 5:**
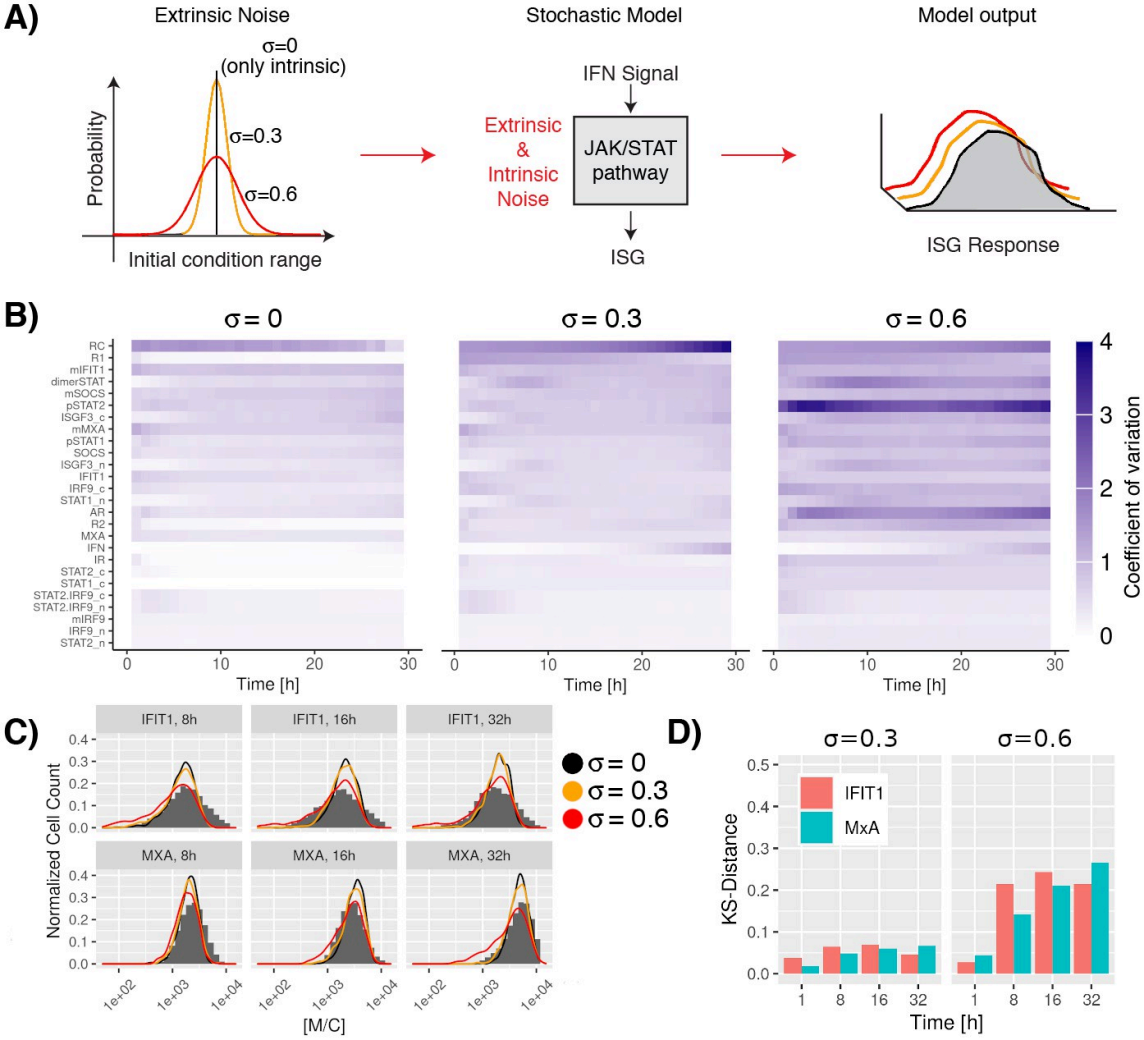
Effect of extrinsic noise in the JAK-STAT signaling pathway. A: Extrinsic noise in the system was introduced by considering the effect of variability in the initial copy number of the proteins in the pathway. Initial conditions were generated by random sampling using a normal distribution $N(\mu ,\;\sigma^{2})$ with values for *μ* given in Table 1 and one of three values of *σ*: 0, 0.3, 0.6. B: Variability in the elements of the JAK-STAT signaling pathway over time. The effects of extrinsic noise in the system were calculated by the coefficient of variation (*cv* = *σ*_*S*_/(*μ*_*S*_ + 0.1), where the subindex *s* represents the species in the pathway). In the plot the colorbar varies between 0 (white color) and larger than 4 (blue color), dark colors represent high variability in the dynamics of the studied species. Species were ordered based on the average coefficient of variation over all time points for systems with an extrinsic noise of *σ* = 0.3. The overall system dynamics under the influence of extrinsic noise can also be observed in Figs J to L in S1 Text. C: Stochastic simulations of different time points after IFN stimulation using a distribution of values as initial conditions. The results show a transient perturbation in the MxA and IFIT1 expression when extrinsic fluctuations are considered. As reference, experimental data for a system without extrinsic noise (*σ* = 0.0) are given (gray histograms). D: Distributions at multiple time points considering different strengths of extrinsic noise *vs* a system with only intrinsic noise are compared using the Kolmogorov-Smirnov distance.

Here, we simply repeated time course simulations many times. Simulations were run for 32 hours and 1,000 repetitions were used to obtain a landscape of the heterogeneity in the population of trajectories. These different trajectories represent the effects of the intrinsic noise and are solely caused by the stochasticity of the individual processes. Our results suggest differences in the temporal responses: species involved in the ligand-receptor activation steps show fast but transient dynamics, whereas elements involved in positive (IRF9) and negative feedback (SOCS) loops display slower and more sustained dynamics (Fig 4B and Fig J in S1 Text). Fig 5B, first panel, depicts (for *σ* equal zero) the intrinsic variation of species over time in a different way. The figure indicates that the highest intrinsic variation lies in the short-lived RC. Otherwise, there is an expected trend that species with low particle numbers show higher variability (for instance SOCS) than species with higher particle numbers (for example STAT1c). However, there are some notable exceptions. Thus, mIRF9 and IRF9 itself which plays an important role in the system show low variability despite the fact that there are only small numbers of the respective molecules around. Of course, such exceptions can be explained by the fact that the stochasticity is not only influenced by particle numbers, but also by reaction rates and their ratios. Therefore, it is noteworthy that the used visualization offers a quick and not completely forseeable overview of the variability of the components in this system.

#### Extrinsic noise in the JAK-STAT signaling pathway

Extrinsic fluctuations can originate from cells undergoing different stages of their cell cycle, and cell-to-cell variability in the copy number of proteins inherited from parent cells during cell division [34]. We focused our attention on the identification of the effect of variability in the copy number of the proteins in the pathway. To this end, we simulated the effect of having a distribution of values as initial conditions instead of fixed values. Initial conditions were generated by random sampling using a normal distribution $N(\mu ,\;\sigma^{2})$ with values for *μ* given in Table 1 and one of three values of *σ*: 0 (again, only intrinsic noise), 0.3, 0.6. By introducing wider distributions as initial condition we tested noisier extrinsic effects in our system (Fig 5A). Subsequently, we quantified the size of the variability in the JAK-STAT signaling pathway by computing the coefficient of variations, which is defined as the ratio of the standard deviation, *σ*_*s*_, to the mean *μ*_*s*_, where the subindex *s* represents the species in the pathway. It is important to remark, that the coefficient of variation is very sensitive to small changes in the mean if the mean value is near zero. Given that there are three species present in the model that are expressed with a copy number of less than one on average (RC, mMxA and mIFIT1), uncertainty in low-probability events plays a major role. Hence, the formula for the coefficient of variations was modified as follows to compensate for high noise caused by low discrete particle numbers:

$$\begin{array}{c} Coefficient\;of\;variation=\sigma_{s}/\left(\mu_{s}+0.1\right) \end{array}$$ (4)

Note, that the effect of the modification is negligible for species with a mean value above one, while statistic uncertainty is reduced for species with a very low average expression.

In contrast to the findings for variability due to intrinsic noise as described above, effects of extrinsic noise are not governed as strongly by the abundance of species. Thus, species that are close to the beginning of the signalling cascade (Fig 5B right column) often show a high level of variability even if they are abundant like pSTAT1 and pSTAT2. The species that constitute the response of the pathway, especially MxA show a lower variability. This is also preseved when increasing the level of extrinsic noise. However, differences in local time-dependent variance are not huge and may not constitute the best way to look at the robustness of the response.

Therefore, we quantified the similarity between responses by measuring the KS-distance between distributions for MxA and IFIT1 under multiple values of extrinsic variability at different time points. Even though, there is clearly an effect of extrinsic noise, the response is conserved with a KS-distance of less than 0.1 for *σ* equal 0.3 and around 0.2 for *σ* equal 0.6 as can be observed in Fig 5C and 5D. In summary, our results indicate that despite variability of individual trajectories, there is an efficient robust transmission of the signal even in noisy environments.

It is noteworthy that fitting of the distribution of the experimental data (Fig 5C) seems to be better if moderate levels of extrinsic noise are added. This observation is in line with previous studies which found that models that incorporate comparable levels of extrinsic noise best resembles their experimental data [83].

#### Parameter robustness in the JAK-STAT signaling pathway

**Fig 6:**
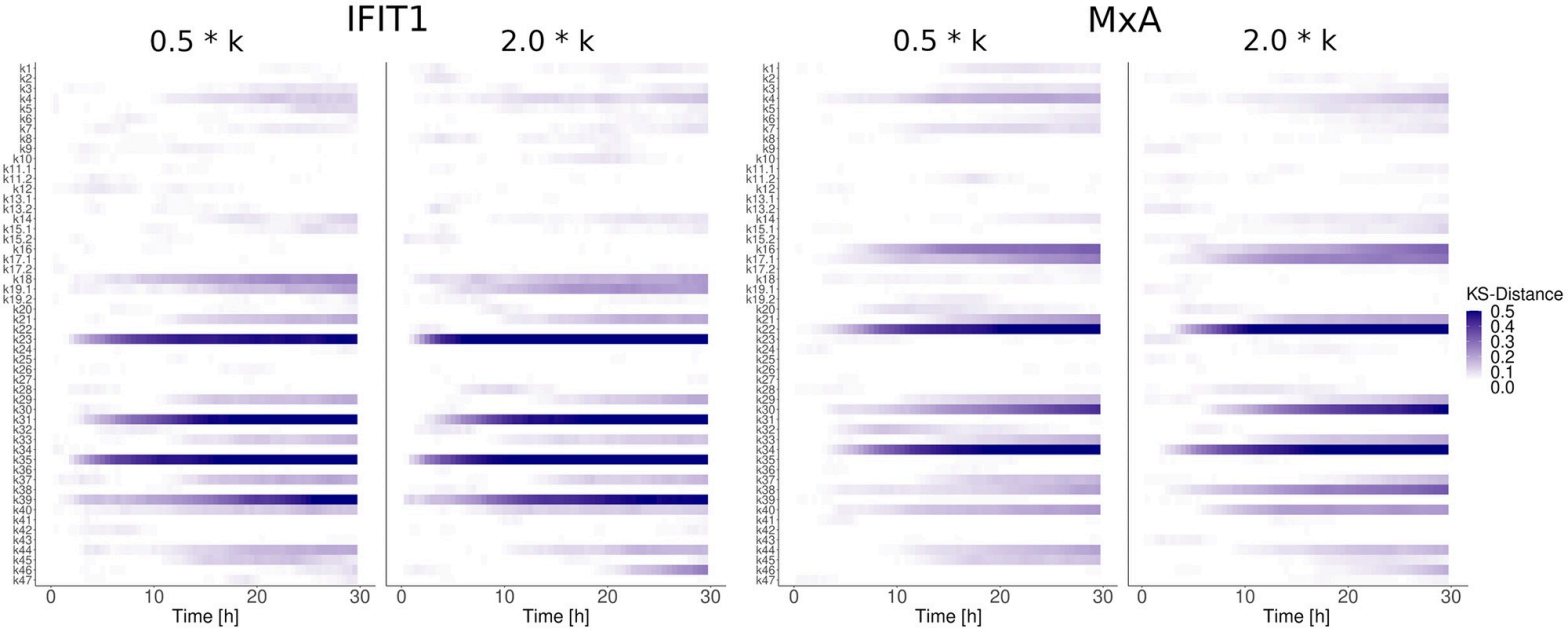
Parameter robustness assay. Parameters were altered between half (0.5 * k) and double (2.0 * k) their original values individually to determine to what extent the evaluated system functionality is preserved under considered perturbations. The effect on the ISG induction was quantified by repeating the stochastic simulation 600 times and computing the KS-distance to the unpertubated system.

**Fig 7:**
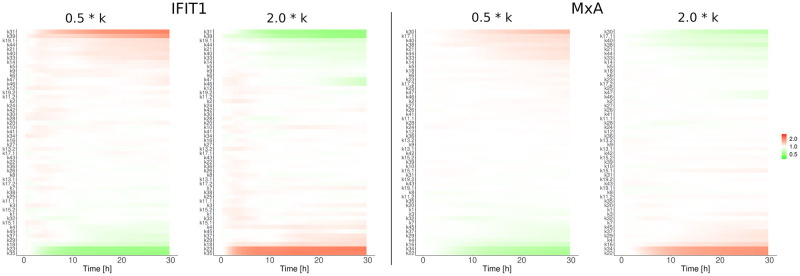
Signal transduction under pertubations. Parameters were altered between half (0.5 * k) and double (2.0 * k) their original values individually to determine which parameters prevent reliable transduction of the signal from receptor to gene expression under considered perturbations. The effect on the ISG induction was quantified by repeating the stochastic simulation 600 times and computing the relative change in IFIT and MxA molecules to the unpertubated system.

To calculate the quality of the obtained parameters it is common practice to repeat the optimization method hundreds or thousands of times until a *posteriori* distribution for each estimated parameter are obtained [84]. This strategy is commonly used for deterministic systems or stochastic systems with a small number of variables. Calculating *posteriori* distributions for the estimated parameter values is used to drive conclusions referent to the sensitivity or robustness of each parameter. In our specific model, given the large number of variables, this strategy is computationally prohibitive. For this reason, we performed a parameter robustness assay to determine to what extent the evaluated system functionality is preserved under considered perturbations (Fig 6). Parameters were altered between half and double their original values individually and after subsequent stochastic simulations, the effect on the ISG induction was quantified using the KS-distance. Given the low KS-distance for all parameter alterations involved in receptor dynamics and signal transduction, it can be assumed that the JAK-STAT signaling pathway is robust to parameter variations. However, higher KS-values have been observed for parameter alterations of parameters involved in the gene expression and degradation of ISGs (transcription, translation, mRNA and protein degradation). Based on the same data, we investigated which parameters prevent reliable transduction of the signal from receptor to gene expression (Fig 7). Again, it was found that perturbing processes in the gene induction (transcription & translation) and decay of the ISGs MxA and IFIT1 altered the average abundance of ISGs while perturbations of other parts of the signal pathway did not cause considerable changes.

## Discussion

During the last two decades, important efforts have been made to increase the understanding of the IFN system by integrating experimental data sets and mathematical models. Existing models of the JAK-STAT signaling pathway describe the deterministic dynamics in cell populations [4, 29–31]. All those efforts neglect the influence of molecular noise in the biochemical reactions taking place along the pathway. Noise has been proven to be a fundamental force affecting multiple cellular pathways, and IFN is no exception. It has been well documented that populations of identical cells produce heterogeneous responses to IFN stimulation [17, 38–41]. The lack of stochastic models to describe the IFN system can be attributed to the scarcity of single-cell data and efficient methods to analyze and make use of this data. Live cell imaging and flow cytometry are among the methods that can be used to measure protein levels in high numbers of single cells. Given that flow cytometry is a relatively easy technique obtaining single-cell measurements of thousands of cells, our approach could be widely translated to other signaling pathways and contexts. Additionally, new methods have recently been introduced to efficiently fit stochastic models to such single-cell data [47, 48]. To the best of our knowledge, we here present the first mechanistic model of the JAK-STAT signaling pathway that is validated with single-cell data and can explain both basal (Fig N in S1 Text) and stimulated states (Fig J in S1 Text).

In summary, we studied the stochasticity of MxA and IFIT1 induction triggered by stimulation of Huh7.5 cells with IFN-*α* for a timescale of 32 hours. To test our working hypothesis, we developed a detailed mathematical model and calibrated it using time-course, single-cell flow cytometry data. This model allowed us to systematically test the influence of intrinsic and extrinsic noise in the IFN response. The major novelty of our model is the fact that it is mechanistic, incorporating the well known architecture of the pathway including major feedback systems and basal states. This contrasts with previous stochastic models that use minimalistic approaches and neglect unstimulated homeostatic states [14]. Unlike previous studies, we present a model that fits two experimental data sets that represent full probability distributions at different time points and immunoblotting measurements. It is important to remark that our model reproduces dynamics within the correct order of magnitude reported for mammalian mRNAs (reported average of 17 Molecules/Cell with range between 1 to 200 Molecules/Cell) and proteins (reported average of 50,000 Molecules/Cell with range between 100 to 10^8^ Molecules/Cell) [71]. Comparing our molecule numbers for STAT1, STAT2 and IRF9-complexes to a more recent publication [65], we can also state that the numbers for STAT1 and STAT2 coincide well while our numbers for IRF9 are lower than measured in this study. However, they correspond to earlier measurements [4] by the same group.

Our results support graded (unimodal) dynamics for MxA and IFIT1. This clearly contrasts with previous reports based on single-cell data for IRF7 (IRF7 is an ISG with a central role in the immune response) where an all-or-none switch response (two subpopulations, responder and non-responder) was observed [14, 15]. We argue that differences in the expression of distinct ISGs may be explained by each ISG promoter architecture. For example, the IRF7 promoter contains two different transcription factor binding sites (ISRE and IRF-E) that are activated by ISGF3 and an IRF7 dimer, respectively. Bimodality in IRF7 expression can be justified by a circuit with a positive feedback loop and the non-linearity caused by the complex activation of its promoter as we showed earlier [48]. On the contrary, our experiments for MxA and IFIT1 show graded responses that were consistently obtained for all IFN doses and multiple time points. MxA and IFIT1 promoters only contain two binding sites for ISGF3 (Section F in S1 Text), and cooperativity has not been proven to take place during type-I IFN responses [85]. The lack of cooperative behavior in the MxA and IFIT1 promoters can explain the observed graded response [85]. It is also tempting to speculate that an all-or-none switch response *vs* graded responses for the ISG induction might be explained by the specific gene regulatory sequences encoded in the gene promoter (Fig M in S1 Text). Moreover, given the simple architecture of their transcriptional sites (only ISREs), very similar MxA and IFIT1 dose-response expression have been observed in different individuals [86, 87]. This suggests that MxA and IFIT1 response to IFN-*α* is robust and explains the graded response that was consistently obtained for all IFN-*α* doses at different time points. Maiwald et al. [4] demonstrated that the dynamic of ISG response and thus the expression of ISGs is largely dependent on the initial concentration of the signal-transduction molecules, which differs both between experimental set-ups and neighboring cells. Hence, contradicting results may be caused by the choice of the cell line, the initial states of the signal-transduction molecules in cells. Finally, we cannot rule out the fact that MxA and IFIT1 graded responses are cell-type specific. Still, we tested a different cell line A549. Our results confirm that MxA and IFIT1 show a graded response upon IFN stimulation and is also consistent with previous reports for MxA [39].

Most initial conditions in the model are assumed to be non-zero before IFN stimulation. This assumption can be justified by previous studies showing a basal expression (around a 10% of the total protein concentration) of phosphorylated forms of STAT proteins [64] and the formation of unphosphorylated STAT2-IRF9 transcription factors [5] even in the absence of IFN stimulation. Additionally, the immunoblotting measurements for IRF9, STAT1 and the active form of the IFN receptor show basal levels of expression even before IFN stimulation [4]. These basal expression levels can be caused by the low and transient enzymatic activities between elements in the pathway [36], or by paracrine responses in the cell culture even before the application of the external IFN stimulus [14].

## Conclusion

By challenging our model we could determine a low intrinsic noise for all species except the receptor complex (RC) and mIFIT1, where the variability is caused by uncertainty in low-probability events. In contrast, high extrinsic noise was observed at the receptor level and during the formation of the transcription factor ISGF3. Nonetheless, extrinsic noise seems to play only a minor role for the JAK-STAT signaling pathway as strong and stable ISG expression was observed for all simulation conditions despite extrinsic noise affecting the dynamics of cellular constituents locally. Additionally, kinetic parameters in the JAK-STAT pathway were found to be resistent to parameter alterations, which supports our hypothesis that the JAK-STAT pathway is a robust system filtering extrinsic noise even further. From a biological point of view this makes perfect sense as multi-cellular organisms depend on a robustly functioning first line of defense against invading pathogens.

## Supporting information

S1 Text Supplementary information. (PDF)
