# Supplementary material for: Stochastic dynamics of Type-I interferon responses
Source: PLoS Comput Biol. 2022 Oct 21;18(10):e1010623. doi: 10.1371/journal.pcbi.1010623 (PMC9629604; doi:10.1371/journal.pcbi.1010623)
Supplement: S1 Text — (PDF) [file pcbi.1010623.s001.pdf]

Supplementary Information - Stochastic Dynamics of Type-I Interferon Responses.

Benjamin D. Maier[1]*, Luis U. Aguilera[1,5]*, Sven Sahle[1], Pascal Mutz[3,4], Priyata Kalra[1], Christopher Dächert[2,4], Ralf Bartenschlager[3,4], Marco Binder[2], Ursula Kummer[1].
[1] Department of Modeling of Biological Processes, COS Heidelberg / Bioquant, Heidelberg University, Heidelberg, Germany.
[2] Research Group "Dynamics of early viral infection and the innate antiviral response", German Cancer Research Center (DKFZ), Heidelberg, Germany.
[3] Division Virus-Associated Carcinogenesis, German Cancer Research Center (DKFZ), Heidelberg, Germany.
[4] Department for Infectious Diseases, Molecular Virology, Medical Faculty, Heidelberg University, Heidelberg, Germany.
[5] Now at: Department of Chemical and Biological Engineering and School of Biomedical Engineering, Colorado State University, Fort Collins, CO, USA

* Equally contributing authors.

Index

A Experimental data **SI-2**
A.1 IFN treatments and Flow cytometry analysis . . . SI-2

B Modeling biological systems **SI-4**
B.1 Stochastic COPASI Model . . . SI-4
B.2 Stochastic modeling . . . SI-7
B.3 Stochastic simulations . . . SI-8
B.4 Statistical moments . . . SI-9

C Parameter estimation strategy **SI-10**
C.1 Integrating experimental data and model dynamics . . . SI-11
C.1.1 Time course data . . . SI-11
C.1.2 Flow cytometry data . . . SI-11
C.2 Comparing deterministic model and cell population data . . . SI-12
C.3 Fitting the stochastic system to flow cytometry data . . . SI-12
C.4 Parameter searches . . . SI-15
C.5 Parameter estimation results . . . SI-17

D Effect of extrinsic noise in the signaling pathway **SI-19**

E Promoter analysis for MxA and IFIT1 **SI-22**

F Basal state **SI-24**

G Effect of nucleus sizes on stochastic dynamics **SI-24**

H Abbreviations **SI-26**

A Experimental data

The data describes the expression of MxA and IFIT1 after IFN-α stimulation in a population of Huh7.5 cells. The experiments were done in the following way: First, cells were transfected with a BAC (Bacterial Artificial Chromosome) containing the studied ISGs (mxa and ifit1) fused with the reporter eGFP genes. Subsequently cultures were treated with different concentrations of human IFN-α. Cells were seeded and left to attach for 24 h. Then, treatment started for 32 h. until all cells including non-treated ones were harvested at the same time point, fixed with 2% PFA to stop further protein expression and applied to flow cytometry. Then, fluorescence in the cultures was monitored using flow cytometry (Figs A and B). We tested the temporal IFN response at time points 8, 12, 16, 20 and 32 hours (Fig C).

A.1 IFN treatments and Flow cytometry analysis

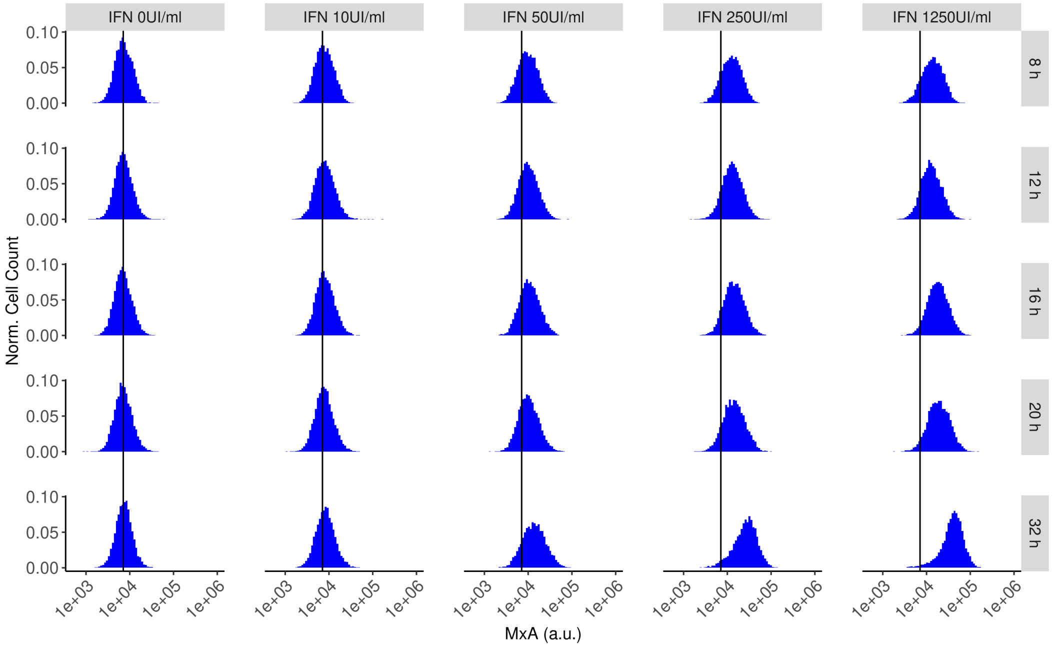

Figure A. MxA flow cytometry data The data shows the expression of MxA after IFN-α stimulation in a population of Huh7.5 cells. Distributions represent the flow cytometry measurements of MxA expression under control conditions (first column, no IFN treatment), subsequent columns represent the MxA expression after multiple IFN doses (from 10 to 1250 UI/mL of IFN-α). In the different IFN treatments, the mean fluorescence level shifts from $8x10^3$ a.u. (arbitrary units of fluorescence) to $4.5x10^4$ a.u. for MxA.

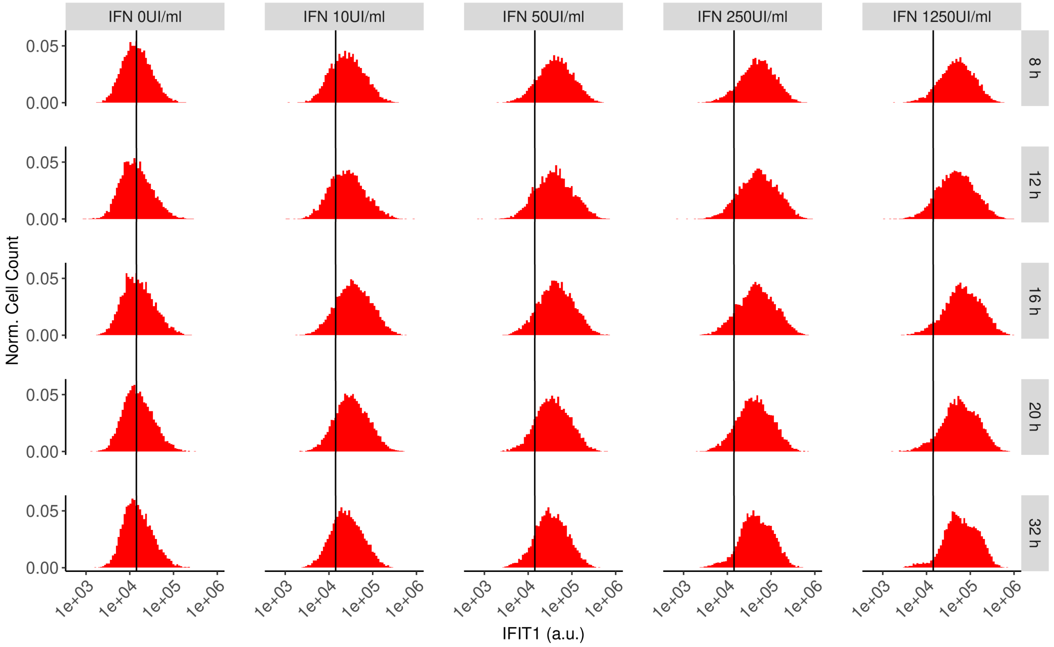

Figure B. IFIT1 flow cytometry data The data describes the expression of IFIT1 after IFN-α stimulation in a population of Huh7.5 cells. Distributions represent the flow cytometry measurements of MxA expression under control conditions (first column, no IFN treatment), subsequent columns represent the MxA expression after multiple IFN doses (from 10 to 1250 UI/mL of IFN-α). In the different IFN treatments, the mean fluorescence level shifts from $2x10^4$ to $9x10^4$ a.u. for IFIT1.

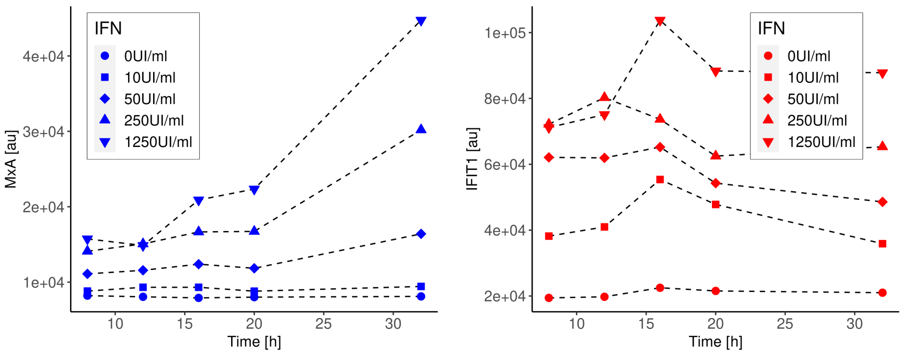

Figure C. Temporal analysis of the IFN response. The plot shows the mean values of the distributions given in Figs A and B. IFIT1 reaches its maximum response 16 hours after IFN stimulation, whereas the maximum response for MxA was obtained after 32 hours.

B Modeling biological systems

B.1 Stochastic COPASI Model

Receptor dynamics.

$$IFN + R2 \xrightarrow{f_1} RC, \tag{M.1}$$

$$RC + R1 \xrightarrow{f_2} AR, \tag{M.2}$$

$$IR \xrightarrow{f_{45}} R1 + R2, \tag{M.3}$$

$$AR + SOCS \xrightarrow{f_{44}} IR + SOCS, \tag{M.4}$$

Receptor-dependent signal transduction.

$$AR + STAT1_c \xrightarrow{f_3} pSTAT1 + AR, \tag{M.5}$$

$$AR + STAT2_c \xrightarrow{f_4} pSTAT2 + AR, \tag{M.6}$$

$$pSTAT1 \xrightarrow{f_5} STAT1_c, \tag{M.7}$$

$$pSTAT2 \xrightarrow{f_6} STAT2_c, \tag{M.8}$$

$$pSTAT1 + pSTAT2 \xrightarrow{f_7} dimerSTAT, \tag{M.9}$$

$$dimerSTAT + IRF9_c \xrightarrow{f_8} ISGF3_c, \tag{M.10}$$

$$ISGF3_c \xrightarrow{f_9} ISGF3_n, \tag{M.11}$$

Receptor-independent signal transduction.

$$STAT2_c + IRF9_c \xrightarrow{f_{10}} STAT2 - IRF9_c, \tag{M.12}$$

$$STAT2 - IRF9_c \xrightarrow{f_{11.1}} STAT2 - IRF_n, \tag{M.13}$$

$$STAT2 - IRF9_n \xrightarrow{f_{11.2}} STAT2 - IRF9_c, \tag{M.14}$$

Promoter activation and inactivation.

$$I\_irf9 + ISGF3_n \xrightarrow{f_{12.1}} irf9, \tag{M.15}$$

$$I\_irf9 + STAT2 - IRF9_n \xrightarrow{f_{12.2}} irf9*, \tag{M.16}$$

$$irf9 \xrightarrow{f_{13.1}} I\_irf9 + ISGF3_n, \tag{M.17}$$

$$irf9* \xrightarrow{f_{13.2}} I\_irf9 + STAT2 - IRF9_n, \tag{M.18}$$

$$I\_socs + ISGF3_n \xrightarrow{f_{14.1}} socs, \tag{M.19}$$

$$I\_socs + STAT2 - IRF9_n \xrightarrow{f_{14.2}} socs*, \tag{M.20}$$

$$socs \xrightarrow{f_{15.1}} I\_socs + ISGF3_n, \tag{M.21}$$

$$socs* \xrightarrow{f_{15.2}} I\_socs + STAT2 - IRF9_n, \tag{M.22}$$

$$I\_mxa + ISGF3_n \xrightarrow{f_{16.1}} mxa, \tag{M.23}$$

$$I\_mxa + STAT2 - IRF9_n \xrightarrow{f_{16.2}} mxa*, \tag{M.24}$$

$$mxa \xrightarrow{f_{17.1}} I\_mxa + ISGF3_n, \tag{M.25}$$

$$mxa* \xrightarrow{f_{17.2}} I\_mxa + STAT2 - IRF9_n, \tag{M.26}$$

$$I\_ifit1 + ISGF3_n \xrightarrow{f_{18.1}} ifit1, \tag{M.27}$$

$$I\_ifit1 + STAT2 - IRF9_n \xrightarrow{f_{18.2}} ifit1*, \tag{M.28}$$

$$ifit1 \xrightarrow{f_{19.1}} I\_ifit1 + ISGF3_n, \tag{M.29}$$

$$ifit1* \xrightarrow{f_{19.2}} I\_ifit1 + STAT2 - IRF9_n, \tag{M.30}$$

Transcription.

$$irf9 \xrightarrow{f_{20.1}} irf9 + mIRF9_n, \tag{M.31}$$

$$irf9* \xrightarrow{f_{20.2}} irf9* + mIRF9_n, \tag{M.32}$$

$$socs \xrightarrow{f_{21.1}} socs + mSOCS_n, \tag{M.33}$$

$$socs* \xrightarrow{f_{21.2}} socs* + mSOCS_n, \tag{M.34}$$

$$mxa \xrightarrow{f_{22.1}} mxa + mMxA_n, \tag{M.35}$$

$$mxa* \xrightarrow{f_{22.2}} mxa* + mMxA_n, \tag{M.36}$$

$$ifit1 \xrightarrow{f_{23.1}} ifit1 + mIFIT1_n, \tag{M.37}$$

$$ifit1* \xrightarrow{f_{23.2}} ifit1* + mIFIT1_n, \tag{M.38}$$

Translocalization / Posttranscriptional Modifications.

$$mIRF9_n \xrightarrow{f_{24}} mIRF9_c, \tag{M.39}$$

$$mSOCS_n \xrightarrow{f_{25}} mSOCS_c, \tag{M.40}$$

$$mMxA_n \xrightarrow{f_{26}} mMxA_c, \tag{M.41}$$

$$mIFIT1_n \xrightarrow{f_{27}} mIFIT1_c, \tag{M.42}$$

mRNA degradation.

$$mIRF9_c \xrightarrow{f_{28}} \varnothing, \tag{M.43}$$

$$mSOCS_c \xrightarrow{f_{29}} \varnothing, \tag{M.44}$$

$$mMxA_c \xrightarrow{f_{30}} \varnothing, \tag{M.45}$$

$$mIFIT1_c \xrightarrow{f_{31}} \varnothing, \tag{M.46}$$

Translation.

$$mIRF9_c \xrightarrow{f_{32}} mIRF9 + IRF9_c, \tag{M.47}$$

$$mSOCS_c \xrightarrow{f_{33}} mSOCS_c + SOCS, \tag{M.48}$$

$$mMxA_c \xrightarrow{f_{34}} mMxA_c + MxA, \tag{M.49}$$

$$mIFIT1_c \xrightarrow{f_{35}} mIFIT1_c + IFIT1, \tag{M.50}$$

Protein degradation.

$$IRF9_c \xrightarrow{f_{36}} \varnothing, \tag{M.51}$$

$$IRF9_n \xrightarrow{f_{36}} \varnothing, \tag{M.52}$$

$$SOCS \xrightarrow{f_{37}} \varnothing, \tag{M.53}$$

$$MxA \xrightarrow{f_{38}} \varnothing, \tag{M.54}$$

$$IFIT1 \xrightarrow{f_{39}} \varnothing, \tag{M.55}$$

$$IFN \xrightarrow{f_{46}} \varnothing, \tag{M.56}$$

Transcription factor release and translocalization.

$$ISGF3_n \xrightarrow{f_{40}} IRF9_n + STAT1_n + STAT2_n, \tag{M.57}$$

$$IRF9_n \xrightarrow{f_{41}} IRF9_c, \tag{M.58}$$

$$STAT1_n \xrightarrow{f_{42}} STAT1_c, \tag{M.59}$$

$$STAT2_n \xrightarrow{f_{43}} STAT2_c, \tag{M.60}$$

$$STAT2 - IRF9_n \xrightarrow{f_{43}} STAT2_n + IRF9_n, \tag{M.61}$$

$$STAT2 - IRF9_c \xrightarrow{f_{43}} STAT2_c + IRF9_c, \tag{M.62}$$

The reaction rates are given in Table A.

Table A. Reaction rates considered in the model

Name	Definition	Name	Definition
f_1	$k_1 \cdot R2 \cdot IFN$	$f_{21.2}$	$k_{21} \cdot socs*$
f_2	$k_2 \cdot RC \cdot R1$	$f_{22.1}$	$k_{22} \cdot mxa$
f_3	$k_3 \cdot STAT1_c \cdot AR$	$f_{22.2}$	$k_{22} \cdot mxa*$
f_4	$k_4 \cdot STAT2_c \cdot AR$	$f_{23.1}$	$k_{23} \cdot ifit1$
f_5	$k_5 pSTAT1$	$f_{23.2}$	$k_{23} \cdot *ifit1$
f_6	$k_6 pSTAT2$	f_{24}	$k_{24} \cdot mIRF9_n$
f_7	$k_7 \cdot pSTAT1 \cdot pSTAT2$	f_{25}	$k_{25} \cdot mSOCS_n$
f_8	$k_8 \cdot dimerSTAT1 \cdot IRF9_c$	f_{26}	$k_{26} \cdot mMxA_n$
f_9	$k_9 \cdot ISGF3_c$	f_{27}	$k_{27} \cdot mIFIT1_n$
f_{10}	$k_{10} \cdot STAT2_c \cdot IRF9_c$	f_{28}	$k_{28} \cdot mIRF9_c$
$f_{11.1}$	$k_{11.1} \cdot STAT2 - IRF9_c$	f_{29}	$k_{29} \cdot mSOCS_c$
$f_{11.2}$	$k_{11.2} \cdot STAT2 - IRF9_n$	f_{30}	$k_{30} \cdot mMxA_c$
$f_{12.1}$	$k_{12} \cdot I\_irf9 \cdot ISGF3_n$	f_{31}	$k_{31} \cdot mIFIT1_c$
$f_{12.2}$	$k_{12} \cdot I\_irf9 \cdot STAT2 - IRF9_n$	f_{28}	$k_{32} \cdot mIRF9_c$
$f_{13.1}$	$k_{13.1} \cdot irf9$	f_{29}	$k_{33} \cdot mSOCS_c$
$f_{13.2}$	$k_{13.2} \cdot irf9*$	f_{30}	$k_{34} \cdot mMxA_c$
$f_{14.1}$	$k_{14} \cdot I\_socs \cdot ISGF3_n$	f_{31}	$k_{35} \cdot mIFIT1_c$
$f_{14.2}$	$k_{14} \cdot I\_socs \cdot STAT2 - IRF9_n$	$f_{36.1}$	$k_{36} \cdot IRF9_c$
$f_{15.1}$	$k_{15.1} \cdot socs$	$f_{36.2}$	$k_{36} \cdot IRF9_n$
$f_{15.2}$	$k_{15.2} \cdot socs*$	f_{37}	$k_{37} \cdot SOCS$
$f_{16.1}$	$k_{16} \cdot I\_mxa \cdot ISGF3_n$	f_{38}	$k_{38} \cdot MxA$
$f_{16.2}$	$k_{16} \cdot I\_mxa \cdot STAT2 - IRF9_n$	f_{39}	$k_{39} \cdot IFIT1$
$f_{17.1}$	$k_{17.1} \cdot mxa$	f_{40}	$k_{40} \cdot ISGF3_n$
$f_{17.2}$	$k_{17.2} \cdot mxa*$	f_{41}	$k_{41} \cdot IRF9_n$
$f_{18.1}$	$k_{18} \cdot I\_ifit1 \cdot ISGF3_n$	f_{42}	$k_{42} \cdot STAT1_n$
$f_{18.2}$	$k_{18} \cdot I\_ifit1 \cdot STAT2 - IRF9_n$	f_{43}	$k_{43} \cdot STAT2_n$
$f_{19.1}$	$k_{19.1} \cdot ifit1$	f_{44}	$k_{44} \cdot AR \cdot SOCS$
$f_{19.2}$	$k_{19.2} \cdot ifit1*$	f_{45}	$k_{45} \cdot IR$
$f_{20.1}$	$k_{20} \cdot irf9$	f_{46}	$k_{46} \cdot IFN$
$f_{20.2}$	$k_{20} \cdot irf9*$	$f_{47.1}$	$k_{47} \cdot STAT2 - IRF9_n$
$f_{21.1}$	$k_{21} \cdot socs$	$f_{47.2}$	$k_{47} \cdot STAT2 - IRF9_c$

B.2 Stochastic modeling

Considering a system of N different chemical species $\mathbf{S} = \{S_1, ..., S_N\}$, the state of the system is defined as the number of molecules of each element in $\mathbf{S}$ at time t: $\mathbf{X}(t) = \big(X_1(t), ..., X_N(t)\big)$. The evolution of the system is given by the interaction of the chemical species through $\mathbf{M}$ reaction channels $\{R_1, ..., R_M\}$. Each reaction channel is represented by the following general scheme:

$$\bar{v}_{1j}X_1 + ... + \bar{v}_{Nj}X_N \rightarrow \hat{v}_{1j}X_N + ... + \hat{v}_{Nj}X_N, \quad (1)$$

for $j = 1, ..., M$, where $v_{ij} = \hat{v}_{ij} - \bar{v}_{ij}$ are the reaction stoichiometries. The stoichiometry matrix $\mathbf{v}$ is composed of all the reaction stoichiometries.

Given the microscopic random processes that govern chemical reactions, it is possible to describe the evolution of $\mathbf{X}(t)$ as a homogeneous Markov process in continuous time. Considering this framework, the Chemical Master Equation (CME) describes the probability that the system has a specific copy number of each S_i at a given point in the future:

$$\frac{\partial P(\mathbf{X}, t)}{\partial t} = \sum_{j=1}^{M} \Big[a_j(\mathbf{X} - v_j)P(\mathbf{X} - v_j, t|X_0, t_0) - a_j(\mathbf{X})P(\mathbf{X}, t|X_0, t_0) \Big] \quad (2)$$

where the two terms within brackets give the rate at which the probability of being in state $\mathbf{X}$ increases or decreases over time because of reactions into or out of state $\mathbf{X}$, respectively. a_j indicates the probability that the reaction R_j will occur in the next infinitesimal interval $[t, t + dt)$.

B.3 Stochastic simulations

Calculating the PDE directly by solving the chemical master equation is in most cases computationally infeasible. Therefore, in this study we approximated the PDE by repeated stochastic simulation runs. Each simulation run results in one realisation of a stochastic time series, and the combined runs let us estimate the evolution of the probabilities of the system being in a certain state at various time points. For reasons of computational efficiency we used an approximate stochastic simulation algorithm, the adapted τ-leap method [1]. In contrast to exact stochastic simulation methods, that simulate each single reaction event, the τ-leap methods estimate the number of reaction events for each biochemical reaction in the model during a suitable chosen time step. We use the method as implemented in the software COPASI [2], which is based on a time step selection scheme and other improvements by Cao et al. [1].
In order to verify the appropriateness of the approximative simulation method, we compared the distributions obtained by 1000 runs of exact simulation using the original direct method [3] with distributions obtained from approximative simulations (which are at least 2 orders of magnitude faster in this model). Fig D shows the differences in the resulting distributions, which are neglectable in the context of this study.

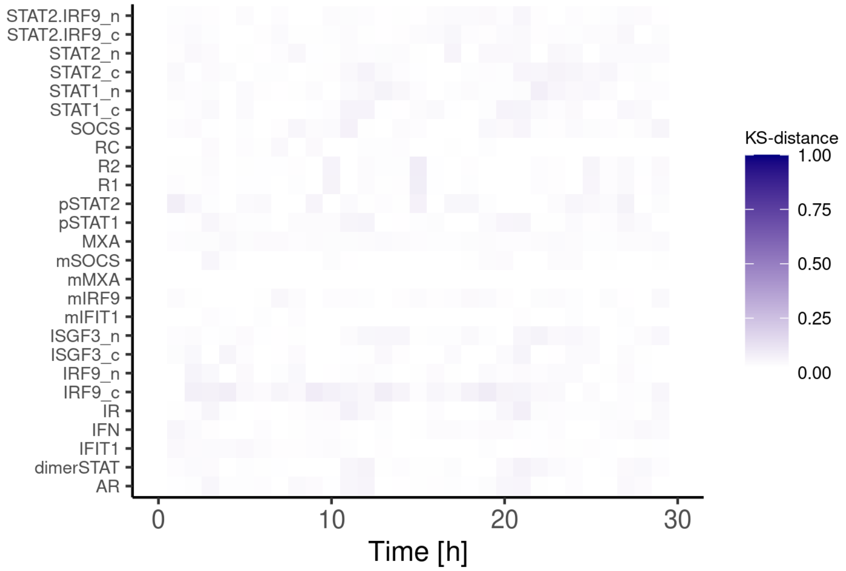

Figure D. Comparison of simulation results using the exact direct method for stochastic simulation vs. the adapted τ-leap method as used in this study. Time dependent PDEs were estimated using 1000 simulations using the exact direct method and the adapted τ-leap method, respectively. The heatmap shows the KS-distance for the two methods at different time points for different variables. The distances are below 0.05 for all variables and timepoints, which is not relevant in the context of this study.

B.4 Statistical moments

Solving the CME or calculating the PDFs from multiple rounds of SSA can give full information regarding the probability of each state at any given time point. Nevertheless, sometimes it is more convenient to collaps the full information to estimate some characteristic properties from those distribution such as the statistical moments [4]. The first statistical moment is known as the mean and is calculated as the sum of each value weighted by the its own probability, that is:

$$\mathbb{E}(X(t)) = \sum_{i=0}^{\infty} X_i P(X_i(t)) \tag{3}$$

The second statistical moment is also known as the variance, and higher moments can be computed, but this will not be discussed in the manuscript.

C Parameter estimation strategy

The aim of the parameter estimation strategy is to find a unique parameter set that can reproduce both the cell population data (time course data from immunoblotting measurements) and the single-cell data (flow cytometry data). Given the different resolutions and time scales in the experimental data sets we divided our optimization strategy in four steps that feedback each other until finding the final parameter values: the first step was a literature search where initial parameter guesses or values ranges were generated, the second step consisted in fitting the model under deterministic dynamics to the cell population data, the third step involved the fitting of the stochastic version of the model with experimental flow cytometry distributions. The final fourth step validates that the stochastic model with the fitted parameter set reproduces both the cell population and single cell data. Concurrently, we estimated the system's initial condition with a similar optimization routine as the basal concentrations for many species incorporated in the model were unknown: Following a literature search to determine or narrow down initial conditions (1), molecule numbers for IRF9_n, mIRF9_c and STAT2-IRF9 complexes as well as localisation distributions of STAT2 were estimated such that the model is in a steady state in absence of interferon under consideration of literature knowledge such as the presence of nuclear retention signals and observed localisation ratios (2). When fitting of the stochastic version of the model with experimental flow cytometry distributions (3), initial ISG particle numbers were assigned to the experimental FACS data of unstimulated cells using scaling factors (OP. 4-7). A diagram of the fitting strategy is given in Fig E. Notice that with this strategy we were able to fit the temporal response of the IFN system and the stochastic dynamics in the final ISG expression.

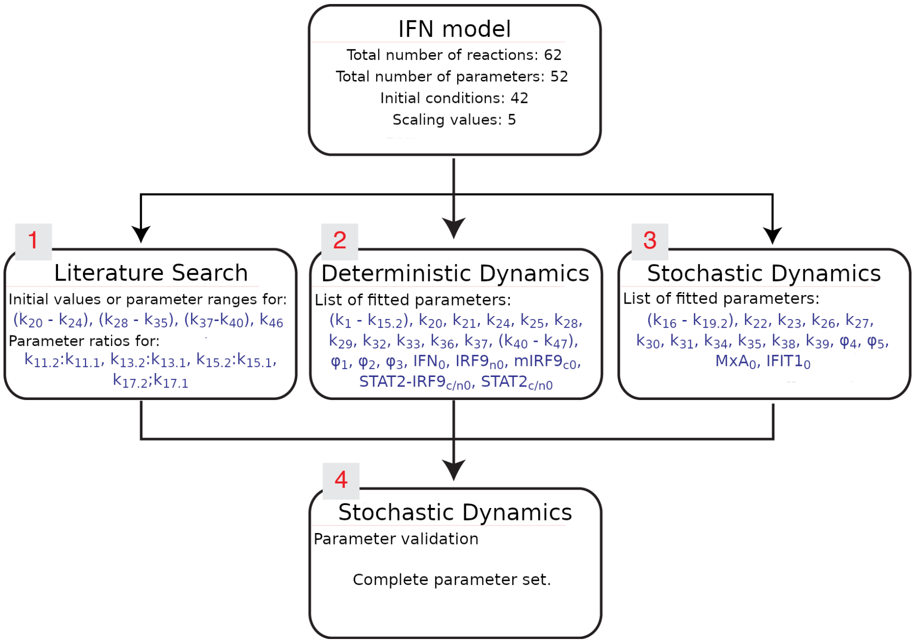

Figure E. Parameter estimation strategy. The parameter estimation strategy was divide in 4 different steps. The figure shows the parameters fitted at each step. The final model reproduces with a single parameter set the cell population and single-cell data. Parameter given in parenthesis represent a range of parameters. The list with the parameter values is given in Table 1, initial conditions are given in Table 3 of the main section.

C.1 Integrating experimental data and model dynamics

C.1.1 Time course data

Time course data were produced by Maiwald *et al.* [5]. The data described the temporal dynamics of different elements of the JAK-STAT signaling pathway. Using quantitative immunoblotting the dynamics of phosphorylated JAK1, pSTAT1 and nuclear IRF9 were measured in Huh7.5 cells after stimulation with 500 UI/mL of IFN-α at different time points for a total time of 180 min. The experimental measurements of phosphorylated JAK1 ($pJAK1^{\dagger}$) were mapped with active receptor (AR) in the model as follows:

$$pJAK1^{\dagger}(t_i) = \varphi_1 * \big(AR(t_i) - AR(t_0)\big), \tag{OP.1}$$

where φ_1 is an scaling factor.

Given that different complexes are detected by the used antibody against phosphorylated STAT1, the experimental measurements of cytoplasmic phosphorylated STAT1 ($pSTAT1^{\dagger}$) were mapped with the different cytoplasmatic complexes of pSTAT1 that are described in the model as follows:

$$\begin{split} pSTAT1^{\dagger}(t_i) = \varphi_2 * \Big(&pSTAT1(t_i) + dimerSTAT(t_i) + ISGF3_c(t_i) \\ &- pSTAT1(t_0) - dimerSTAT(t_0) - ISGF3_c(t_0)\Big), \end{split} \tag{OP.2}$$

where φ_2 is an scaling factor.

The experimental measurements of nuclear IRF9 ($IRF9^{\dagger}$) were mapped with the nuclear complexes involving IRF9 in the model as follows:

$$\begin{split} IRF9^{\dagger}(t_i) = \varphi_3 * \Big(&ISGF3_n(t_i) + STAT2\text{-}IRF9_n(t_i) + IRF9_n(t_i) \\ &- ISGF3_n(t_0) - STAT2\text{-}IRF9_n(t_0) - IRF9_n(t_0)\Big), \end{split} \tag{OP.3}$$

where φ_3 is an scaling factor.

C.1.2 Flow cytometry data

Experimental data describing expression of MxA and IFIT1 after IFN-α stimulation in a population of Huh7.5 cells. Experiments were done in the following way: first, cells were transfected with a BAC (Bacterial Artificial Chromosome) containing MxA and reporter GFP and dGFP genes fused, subsequently cultures were treated with two concentrations of IFN-α (100 and 1250 UI/mL). For illustrative purposes we selected the treatment with 250 UI/mL of IFN-α. Then, fluorescence in the cultures was monitored using flow cytometry at different time points during 32 hours.

The experimental measurements of MxA ($MxA^{\dagger}$) were mapped with the MxA in the model as follows:

$$MxA^{\dagger}(t_i) = \varphi_4 * MxA(t_i), \tag{OP.4}$$

where φ_4 is an scaling factor.

The experimental measurements of IFIT1 ($IFIT1^{\dagger}$) were mapped with the $IFIT1$ in the model as follows:

$$IFIT1^{\dagger}(t_i) = \varphi_5 * \big(IFIT1(t_i) + 65\big), \tag{OP.5}$$

where φ_5 is an scaling factor and 65 serves as numeric constant to compensate for IFN-independent IFI1 expression through cross-talk (e.g. STAT1-independent Trans-activation of ISG56 promoter by IRF-3 [6, 7]). Even though MxA is also affected by IFN-independent cross-talks, no compensation was applied as its expression differs from IFIT1 in being dependent on STAT1 signaling [7,8] and due to measurements indicating that IFN scores correlate strongly with monocyte MxA proteins [9, 10]

The initial particle numbers of MXA and IFIT1 of the model given in Table 3 of the main section were calculated as follows:

$$MxA(t_0) = mean\big(MxA^{\dagger}(IFN_0)\big)/(\varphi_4 * V_{Cytoplasm}), \tag{OP.6}$$

$$IFIT1(t_0) = mean\big(IFIT1^{\dagger}(IFN_0)\big)/(\varphi_5 * V_{Cytoplasm}), \tag{OP.7}$$

where φ_4 and φ_5 are scaling factors. Note that for the stochastic simulations the reporter's initial conditions were sampled from a log-normal distribution which adequately reproduces experimental data (Fig F) rather than taking the scaled experimental mean expression.

Table B. Scaling factors

Scaling Factor	Value
φ_1	1.14×10^{-3}
φ_2	5.43×10^{-5}
φ_3	1.07×10^{-3}
φ_4	5.099
φ_5	29.62

C.2 Comparing deterministic model and cell population data

The measurements of time course data represent the average dynamics of proteins in a population of Huh7.5 cells. Those measurements were related to the the corresponding observable chemical species in the model with a specific set of parameter values $\theta = \{\theta_1, ..., \theta_d\}$, using a squared differences functional:

$$F_D(\theta, S^O) = \sum_{i=1}^{m}\sum_{j=1}^{n}(S_{ij}^{\dagger} - S_{ij}^{O}(\theta))^2. \tag{OP.8}$$

C.3 Fitting the stochastic system to flow cytometry data

Flow cytometry is a high-throughput technology that measures single-cell fluorescence from labeled biomolecules through a detector system. By an automated process, it measures thousands of cells at a time capturing in this way the cell-to-cell variability in the culture. Measurements of the same cell population at different time points can be taken and the temporal evolution of the whole population can be monitored. Given the resolution obtained by flow cytometry and the large number of repetitions it is an excellent source of data to fit and analyze stochastic models.

The process to fit the stochastic system to flow cytometry data was developed based on Lillacci's [11] and Aguilera's works [12]. Commonly, flow cytometry measurements are analysed by histograms or probability density functions (PDFs). However, a drawback of theese representations is that their shape is dependent on the number of bins used for its construction. For this reason, cumulative density functions (CDFs) have been

suggested as more accurate representations of flow cytometry data [13]. The CDF of a random variable x is the probability that the random variable is less than or equal to some value, that is: $F(a) = P(x \leq a)$ [13].

In our case, we build empirical CDF (ECDF) for experimental data and simulations results. First, having nm repetitions of single-cell experimental data from flow cytometry measurements at I time points t_i, i = 1, . . . , I, that is $\boldsymbol{m}(t_i) = \{m_1(t_i), ..., m_{nm}(t_i)\}$ ECDFs for the experimental data $\hat{F}_e(\boldsymbol{m}(t_i))$ were built. In a similar way, considering a specific set of parameter values $\theta = \{\theta_1, ..., \theta_d\}$, we performed ns repetitions of the stochastic simulations $\boldsymbol{s}(t_i) = \{s_1(t_i), ..., s_{ns}(t_i)\}$. The total of those stochastic simulations were used to build the ECDF for each t_i that is $\hat{F}_s(\boldsymbol{s}(t_i), \theta)$.

To calculate the distance between $\hat{F}_e$ and $\hat{F}_s$ we used the Kolmogorov distance (D_{KS}), that is the absolute difference between two ECDFs [13]. For $\hat{F}_e$ and $\hat{F}_s$ their Kolmogorov distance is:

$$D_{KS} = \max_x |\hat{F}_e - \hat{F}_s| \qquad \text{(OP.9)}$$

Experimental data distributions came from measurements of tens of thousands of single cells by flow cytometry, whereas distributions from the stochastic model requires ns stochastic simulations. Computing the model distribution using ns in the order of tens of thousands is computationally expensive even for simple models. To reduce computational cost in our simulations we calculated a minimal number of stochastic simulations $\hat{ns}$ needed to build the distribution with a quality good enough to be used during the optimization strategy. An important finding introduced by Lillacci [11,13] is the definition of a minimal number of simulations $\hat{ns}$ needed to apply the Kolmogorov distance. It is calculated using the properties of the Kolmogorov distribution as follows:

$$\hat{ns} = \left\lceil \frac{-log(\frac{\alpha}{2})}{2\left(\epsilon - \sqrt{-\frac{1}{2 \cdot nm} log\frac{\alpha}{2}}\right)^2} \right\rceil, \qquad \text{(OP.10)}$$

where $\lceil x \rceil$ represents the closest integer to x. $\alpha = 1 - \sqrt{1-\beta}$ and β represent a fixed confidence level, ϵ represents the desired tolerance [13]. In Lillacci's implementations typical values for $\beta = 0.05$ (representing 95% confidence) and $\epsilon = 0.05$ are used.
Since the experimentally obtained flow cytometry measurements suggest a logarithmic normal distribution of protein concentrations in the unstimulated state, initial conditions were sampled from a log-normal distribution (Fig F).

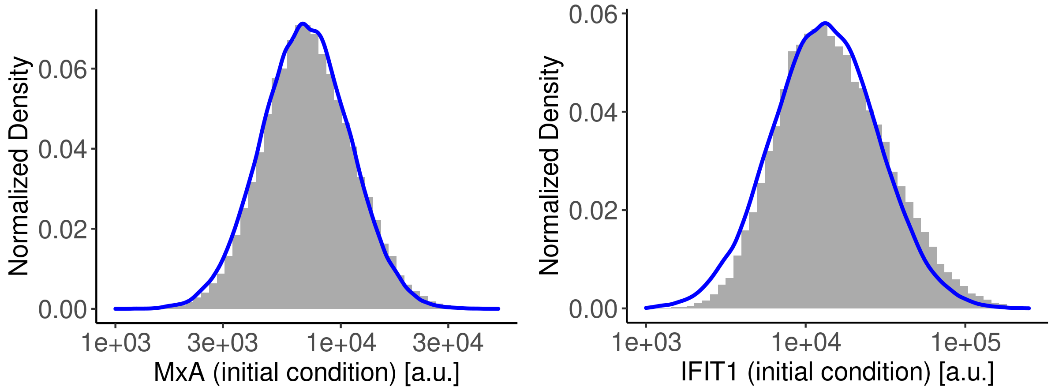

Figure F. The reporter's initial conditions were sampled using a log-normal distribution (blue line) which adequately reproduces experimental data (histogram).
Time series data of all flow cytometry measurements of unstimulated cells (0 UI/mL IFN) were combined and its mean value and standard deviation calculated to determine the formula of the logarithmic normal distribution for basal expression. The lognormal distribution for MxA is characterized by a log mean value of 8.85 and a a standard deviation on the log scale of 0.45, while IFIT1's initial particle number is sampled from a lognormal distribution with a log mean of 9.48 and a log sd of 0.45. The IFIT initial particle number was adjusted with 65 molecules times scaling factor 5 to adress for IFIT1 expression through cross-talk (see Section S3.1.2). The Kolmogorov-Smirnov distance $D_K S$ between experiment and log-normal distribution is below 0.05. In the plot, the x-axis represents the fluorescence level in arbitrary units of fluorescence. The integral over the density (area under the curve) is normalized so that it equals one. Prior to that, it was tested whether the conditions for assuming a normal distribution were given.

C.4 Parameter searches

Parameter searches consisted in optimization routines based on genetic algorithms (GA) [14, 15]. GAs mimic evolution and are based on the mutation, reproduction and selection. By the continuous process of selecting the best parameters after each generation, the algorithm evolves towards a minimum in parameter space. Our optimization strategy is based on Aguilera *et al.* [12]. The proposed method improves its performance by selecting parameters values after comparing the similitude between the first statistical moment of the system and the first statistical moment in the experimental data distribution. By this pre-selection of parameter values most of the original parameters are rejected and the algorithms focus on the finding of parameters that reproduce the observed distribution dynamics. This pre-step significantly reduces the computational cost. For our optimization routine we implemented an population of 1000 individuals for 15 generations. The following settings for genetic operators were chosen: random (log-uniform) population of real values, linear-rank selection, single-point crossover and log-uniform random mutation. As parameters for the algorithm we used an elitism rate $\upsilon = 0.2$, a crossover rate of 0.8 and a mutation rate $\mu = 0.2$. At the end of the generations the best solution of the algorithm was selected as θ_{fit}. A pseudo-code for the GA is given in Algorithm S2 and a graphical description is given in Fig G.

Data: High-throughput PDFs. Biochemical Model.
Define: Number of Free Parameters (θ_{fp}). Ranges for Parameter Values. Number of Generations (G). Population Size (PS). Mutation Rate (μ). Rate of Elitism (υ).
Result: Parameter values that best reproduce the experimental data.

GENERATE a initial population of random parameters.

for $i = 1 : G$ **do**
  $j = 1$;
  while $j < PS$ **do**
    Assign the jth parameter set in the model;
    Run deterministic dynamics ;
    Test deterministic precondition ;
    if *deterministic precondition is true* **then**
      RUN stochastic simulations ;
      Objective Function (OF) evaluation ;
      $fitness = -OF$
    else
      Reject the jth parameter set ;
      Set OF = deterministic evaluation value ;
      $fitness = -OF$;
    end
    $j = j + 1$
  end

  RANK individuals according to its $fitness$;
  SELECT a number of parental individuals (PI), $np = PS \times \epsilon$;
  RECOMBINE PI until generate a offspring number, $no = PS - PI$;
  MUTATE each offspring with a number of mutations, $nm = \theta_{fp} \times \mu$;

end

Algorithm S1: Genetic algorithm with a deterministic precondition

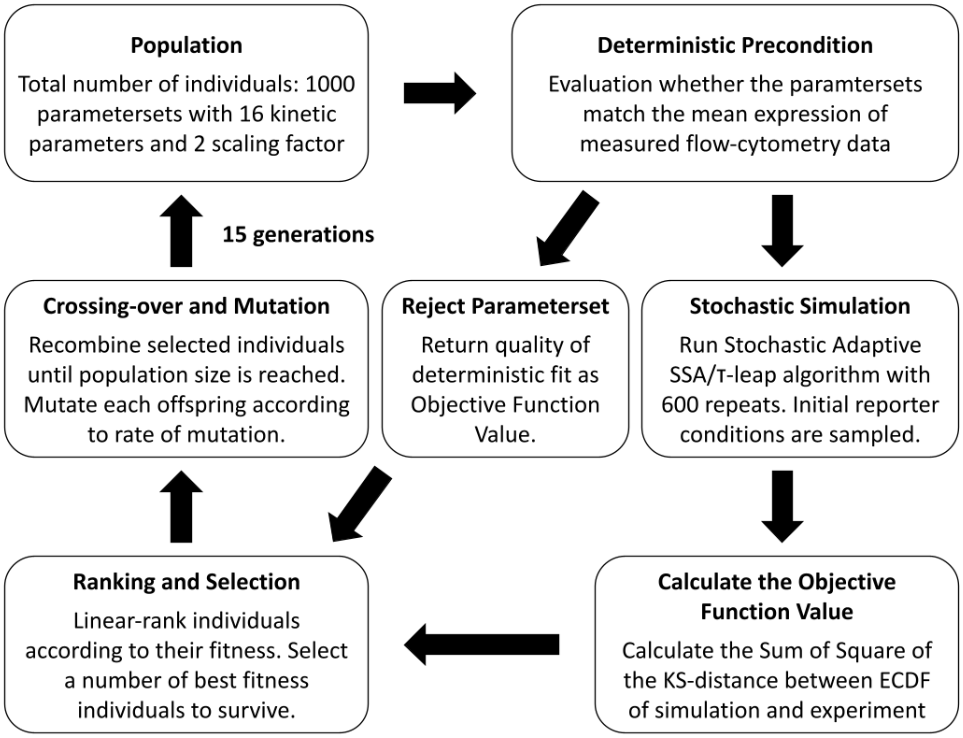

Figure G. Genetic algorithm strategy with deterministic preconditions. Genetic algorithms are stochastic search algorithms that resemble natural selection and sexual reproduction by mimicking the biological mechanisms of selection, recombination and mutation. This algorithm is made of a population of individuals (parameter sets), and each contains a genome that is defined by the number of parameters to optimize. The individuals are ranked after solving the objective function, and a population of parental individuals is selected according to an elitism rate (υ). New individuals (offspring) are generated by pairing and recombining the parental genomes (cross-over). Variability is introduced in the population by adding mutations in the new individuals according to a given mutation rate (μ). By the continuous process of selecting the best parameters after each generation, the algorithm evolves towards a minimum in parameter space. Our optimization strategy is based on Aguilera *et al.* [12]. The proposed method improves its performance by selecting parameters values after comparing the similitude between the first statistical moment of the system and the first statistical moment in the experimental data distribution. By this pre-selection of parameter values most of the original parameters are rejected and the algorithms focus on the finding of parameters that reproduce the observed distribution dynamics. This pre-step significantly reduces the computational cost.

C.5 Parameter estimation results

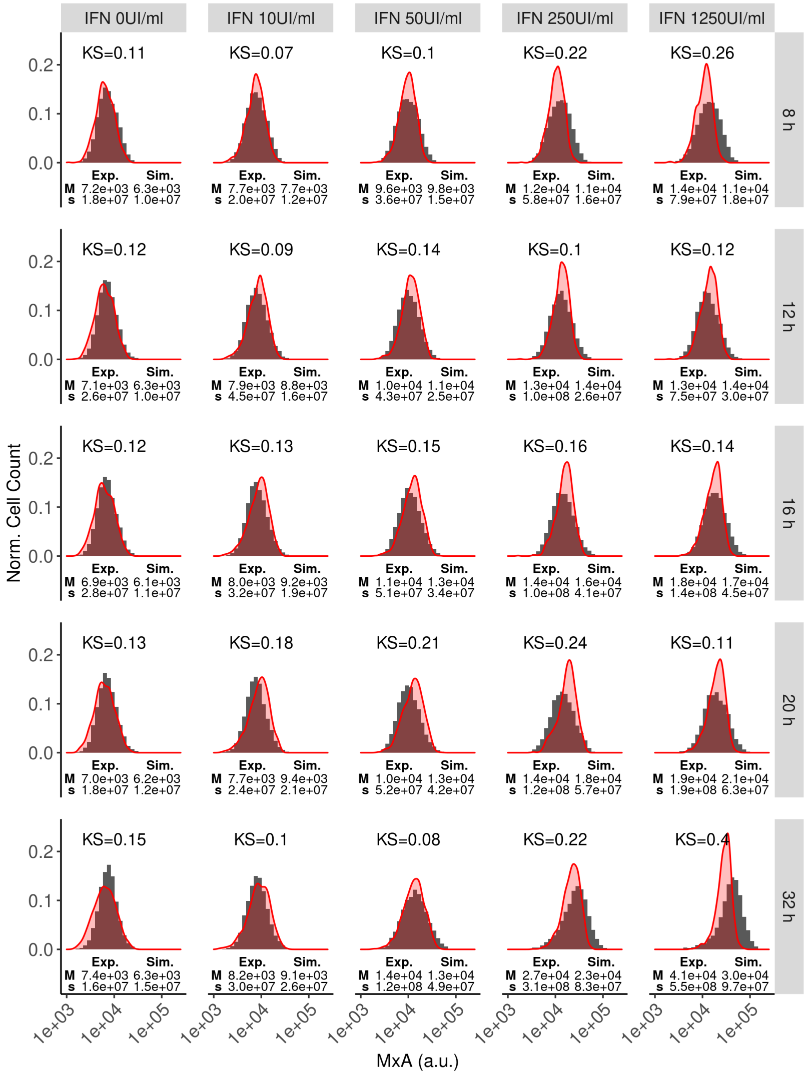

Figure H. Fitted MxA reporter single-cell data to the stochastic model. The parameterized model can fully capture the heterogeneity in the IFN response for the surrogate marker MxA at five different time points upon stimulation with various IFN concentrations ranging from 0 – 1250 UI/mL. For each distribution, the median (M) and variance (s) are given. A switch-like expression is observed for single-cell trajectories, whilst the whole population displayed unimodality. At the higher IFN concentrations, our model underestimates the variance of the distributions.

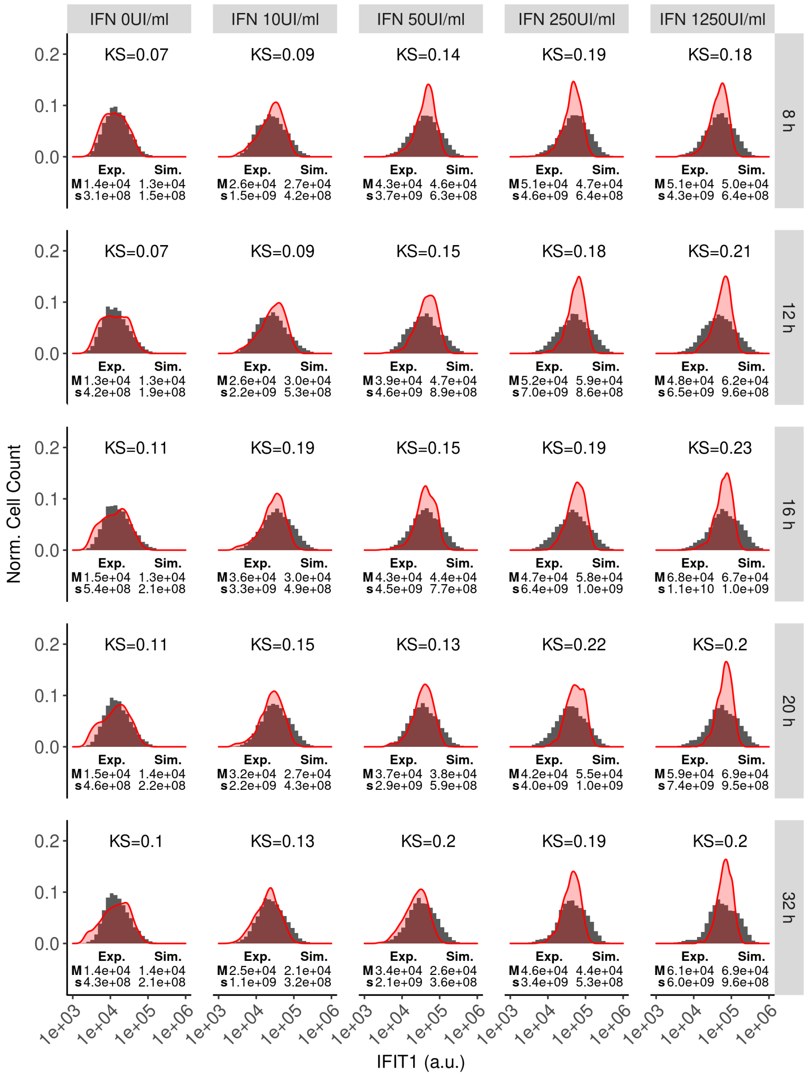

Figure I. Fitted IFIT1 reporter single-cell data to the stochastic model. The parameterized model can fully capture the heterogeneity in the IFN response for the surrogate marker MxA at five different time points upon stimulation with various IFN concentrations ranging from 0 – 1250 UI/mL. For each distribution, the median (M) and variance (s) are given. A switch-like expression is observed for single-cell trajectories, whilst the whole population displayed unimodality. Again, our model underestimates the variance at higher IFN concentrations.

D Effect of extrinsic noise in the signaling pathway

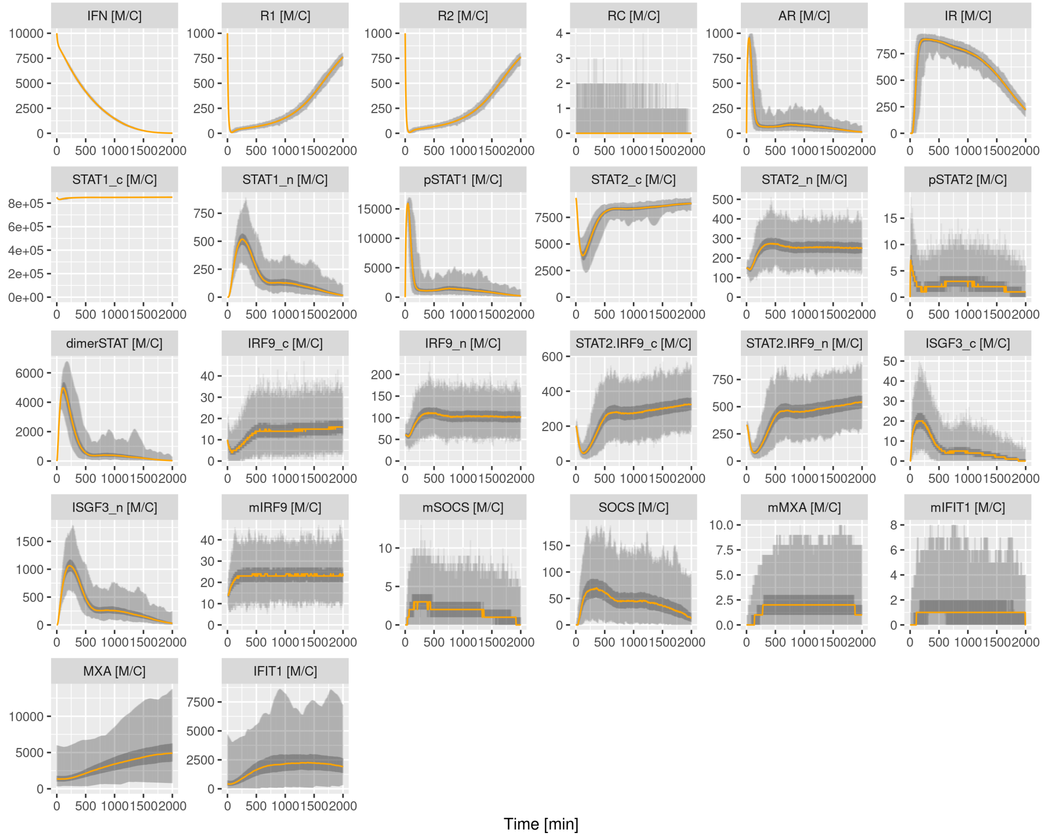

Figure J. Model temporal stochastic dynamics without extrinsic noise. Time course data describing the temporal dynamics of all species involved in the JAK-STAT signaling pathway. The plots represent the median values calculated with the repetitions of the stochastic model (orange lines). The y-axis has units of Molecules per Cell (M/C). The range of the distributions is indicated by the light gray ribbons, while dark gray ribbons represent 50% KI intervals.

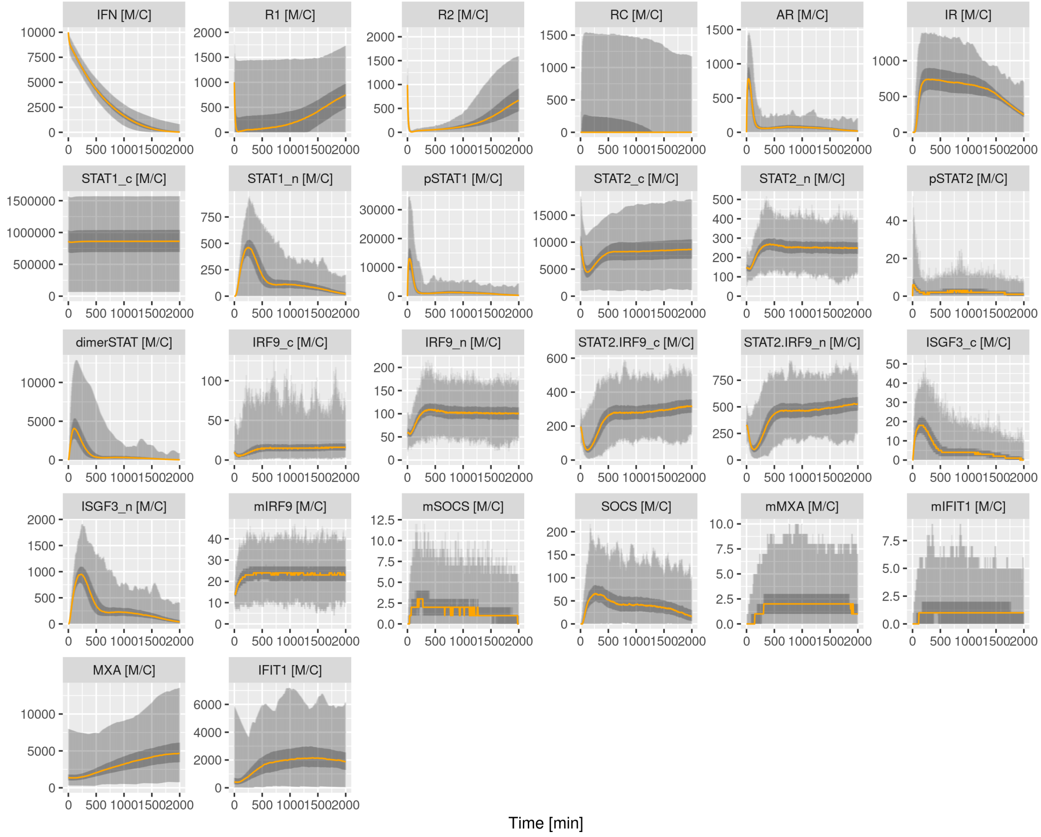

Figure K. Model temporal stochastic dynamics for a system with extrinsic noise ($\sigma = 0.3$).
Time course data describing the temporal dynamics of all species involved in the JAK-STAT signaling pathway. The plots represent the median values calculated with the repetitions of the stochastic model (orange lines). The y-axis has units of Molecules per Cell (M/C). The range of the distributions is indicated by the light gray ribbons, while dark gray ribbons represent 50% KI intervals.

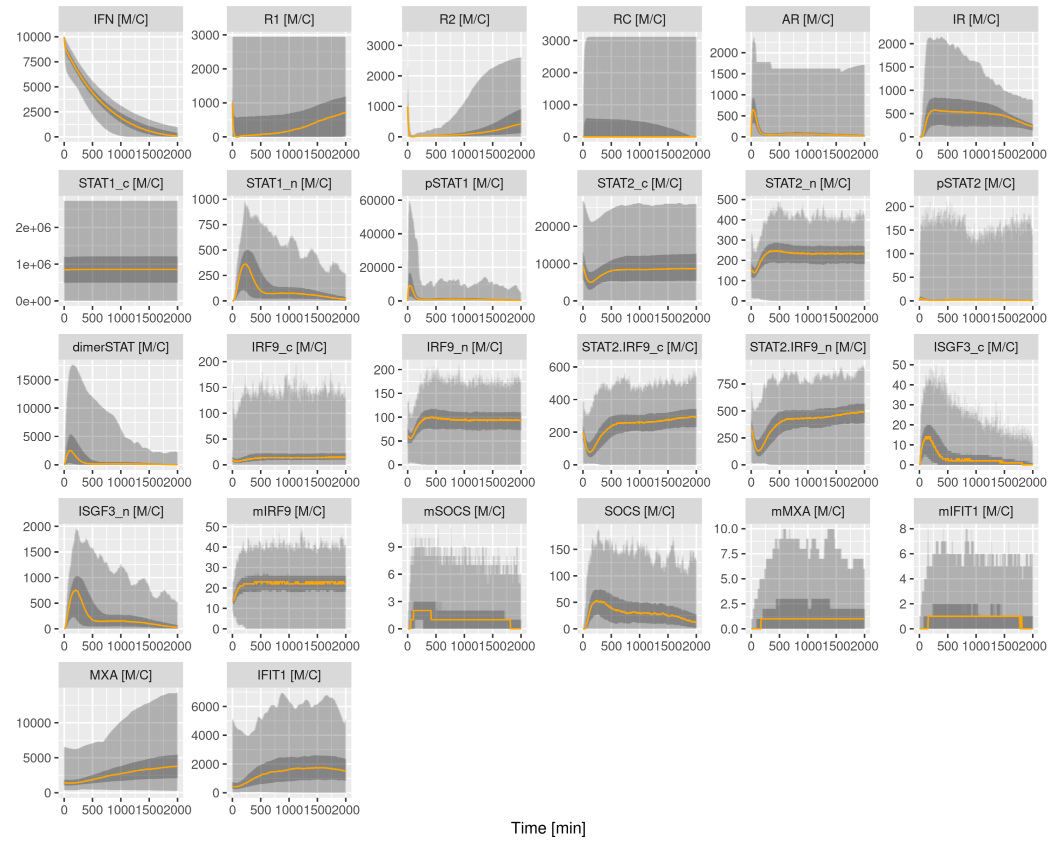

Figure L. Model temporal stochastic dynamics for a system with extrinsic noise ($\sigma = 0.6$).
Time course data describing the temporal dynamics of all species involved in the JAK-STAT signaling pathway. The plots represent the median values calculated with the repetitions of the stochastic model (orange lines). The y-axis has units of Molecules per Cell (M/C). The range of the distributions is indicated by the light gray ribbons, while dark gray ribbons represent 50% KI intervals.

E Promoter analysis for MxA and IFIT1

MxA promoter sequence

MxA promoter contains two functional IRES sites (marked in red in the following sequence) that have been experimentally proved to be activated by ISGF3. [16, 17].

TCATCAGTTAAGGCTGTTTTTACTTCTTTTGTGGATCTTCAGTTACTTTAGGCCATCTGGATGTATACCTGCAAGTC
ACAGGGGATGCGATGGCCTGGCCTGGGATGCGATGGCCTGGCCTGACAACTATTACCTATGTTATGTTTATTATTTT
AAGCTTTATTATTACTATTTTATTTATTTTATTTTATTTTCCTTCCACACACCCGTTTCCACCCTGGAGAGGCCAGAT
GAGCCAGACTCCAGGGAGGCCTAGAAGTGGGCAAGGGGAAACGGAAAGGAGGAAGATGGTATGGGTGTGCCTGGT
TAGGGGTGGGAGTGCTGGACGGAGTTCGGGACAAGAGGGGCTCTGCAGCCATTGGCACACAATGCCTGGGAGTCCC
TGCTGGTGCTGGGATCATCCCAGTGAGCCCTGGGAGGGAACTGAAGACCCCCAATTACCAATGCATCTGTTTTCAAA
ACCGACGGGGGGAAGGACATGCCTAGGTTCAAGGATACGTGCAGGCTTGGATGACTCCGGGCCATTAGGGAGCCTC
CGGAGCACCTTGATCCTCAGACGGGCCTGATGAAACGAGCATCTGATTCAGCAGGCCTGGGTTCGGGCCCGAGAAC
CTGCGTCTCCCGCGAGTTCCCGCGAGGCAAGTGCTGCAGGTGCGGGGCCAGGAGCTAGGTTTCGTTTCTGCGCCCG
GAGCCGCCCTCAGCACAGGGTCTGTGAGTTTCATTTCTTCGCGGCGCGGGGCGGGGCTGGGCGCGGGGTGAAAGAG
GCGAAGCGAGAGCGGA

IFIT1 promoter sequence

IFIT1 promoter contains two functional IRES sites (marked in red in the following sequence) that have been experimentally proved to be activated by ISGF3. [18].

TTTTAGACGGAGTCTCGCTCTGTCACCAGACTGGAGTACAGTGGTGTGATCTCGGCTCACTGCAACCTCTGCCTCC
CAGGTTCAAGCAATTCCCCTGCCTCAGCCTCTCGAGTAGGTGGGACTACAGGTGCACACCACCACACCCAGCTAAT
TTTTTGTATTTTAGTAGAGAGGGGGTTTCACCATGTTGGCCACGATGGTCTCCATCTCCTGACCTTGTCATCCGCC
CACCTTGGCCTCCCAAAGTGCTGGGACTACAGGCATGAGCCACCGCACCCAGCCAAGAATCATTATTTTTAACTTG
ATGACTGAAAATAATAATAATAATAGTTACCACTTATTTGCATGCTTCTATGTGCCAGGTAGTTGCTAACTATTTA
AACTCAAATTCCATGAACTGTAGTGGAGGTTGTACTGGAATTTGATTCAGAATGACAGTGTCCATGATGGAGCAAT
AGAGGGCTCTCTATTTCAAACCATACCTCCTTGCTTTTACCTCCTGCCTAAGTCATCAGGGGTTAGAAGGCTTTCT
AGGTATTGGTCTCTTTCCTTCATTCCTAAACCAGATTGGTTGCTTATTTCCGTCAAGCTGAAACCAAAAGTAAGCA
ACCAAAAAGCAACCAGCAACCAAAAGCCTTGTTACTCAATTAATTAAGAGTAGATTTTTATATTTGATAGTAGGTT
CCTTCTAAATATAGAAACTGAAAATAGAGCTATCTCCTTCAATTCTCCTTTTTCTGTGTATTCATCCAGAATCCAG
CCACCAACTGCCACAATAGGCAGCAATGGACTGATGTTCTTTAGGGAGGACGTGAATCTCGTTCCAAATGCTGGCC
AGTCATTGGGTTTCTGCAGCACTAGAAACATCTATGGTTGCAGGTCTGCAGTTTATCTGTTTTAAAATAGAAACAA
AGTTTCATTCCCCACCCCCCCCCGTCAGCAGGAATTCCGCTAGCTTTAGTTTCACTTTCCCCTTTCGGTTTCCCTAGG
TTTCCAACTT

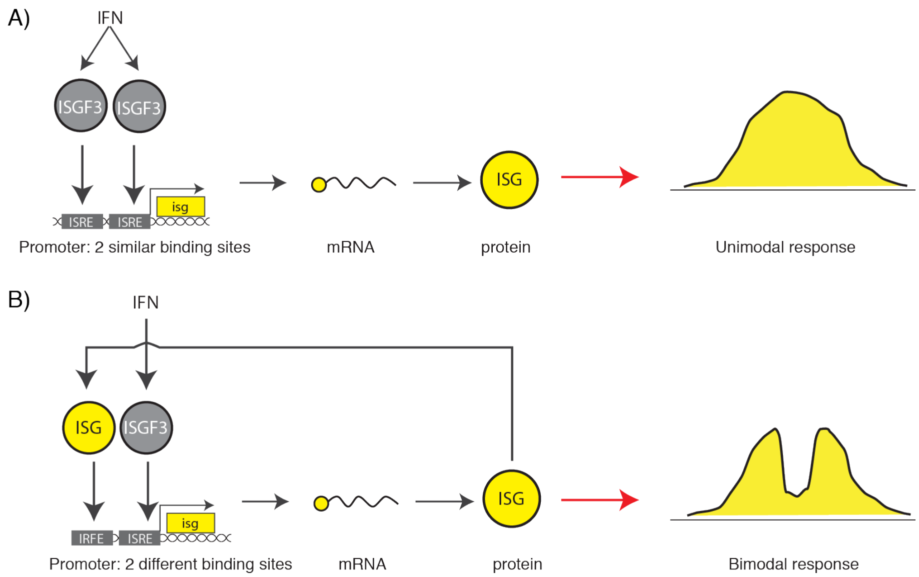

Figure M. ISG promoter architectures and gene expression dynamics. Different promoter architectures in the ISG may explain the particular IFN response. **A)** MxA and IFIT1 promoters only contain two transcription binding sites for ISGF3, and cooperativity has not been proved to take place during IFN type-I responses [18]. The lack of cooperative behavior in the MxA and IFIT1 promoters can explain the observed graded (unimodal) response. **B)** IRF7 promoter contains two different transcription factors binding sites (ISRE and IRF-E) that are activated by ISGF3 and a IRF7 dimer, respectively. Bimodality (all-or-none switch response) in IRF7 expression can be justified by circuit with a positive feedback loop and the non-linearity caused by the complex activation of its receptor [12, 19].

F Basal state

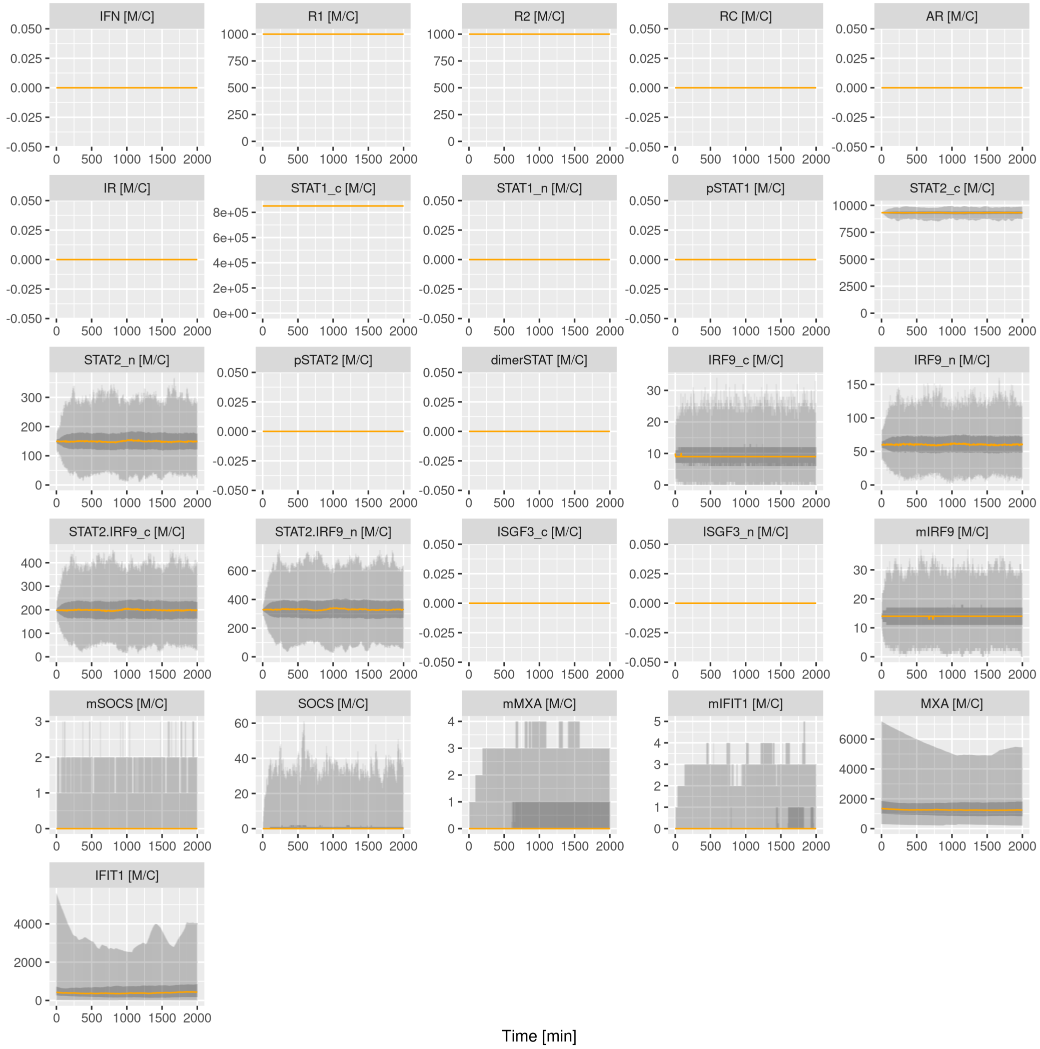

Figure N. Model temporal stochastic dynamics for a system without IFN treatment (basal state).
Time course data describing the temporal dynamics of all species involved in the JAK-STAT signaling pathway. The plots represent the median values calculated with the repetitions of the stochastic model (orange lines). The y-axis has units of Molecules per Cell (M/C). The range of the distributions is indicated by the light gray ribbons, while dark gray ribbons represent 50% KI intervals.

G Effect of nucleus sizes on stochastic dynamics

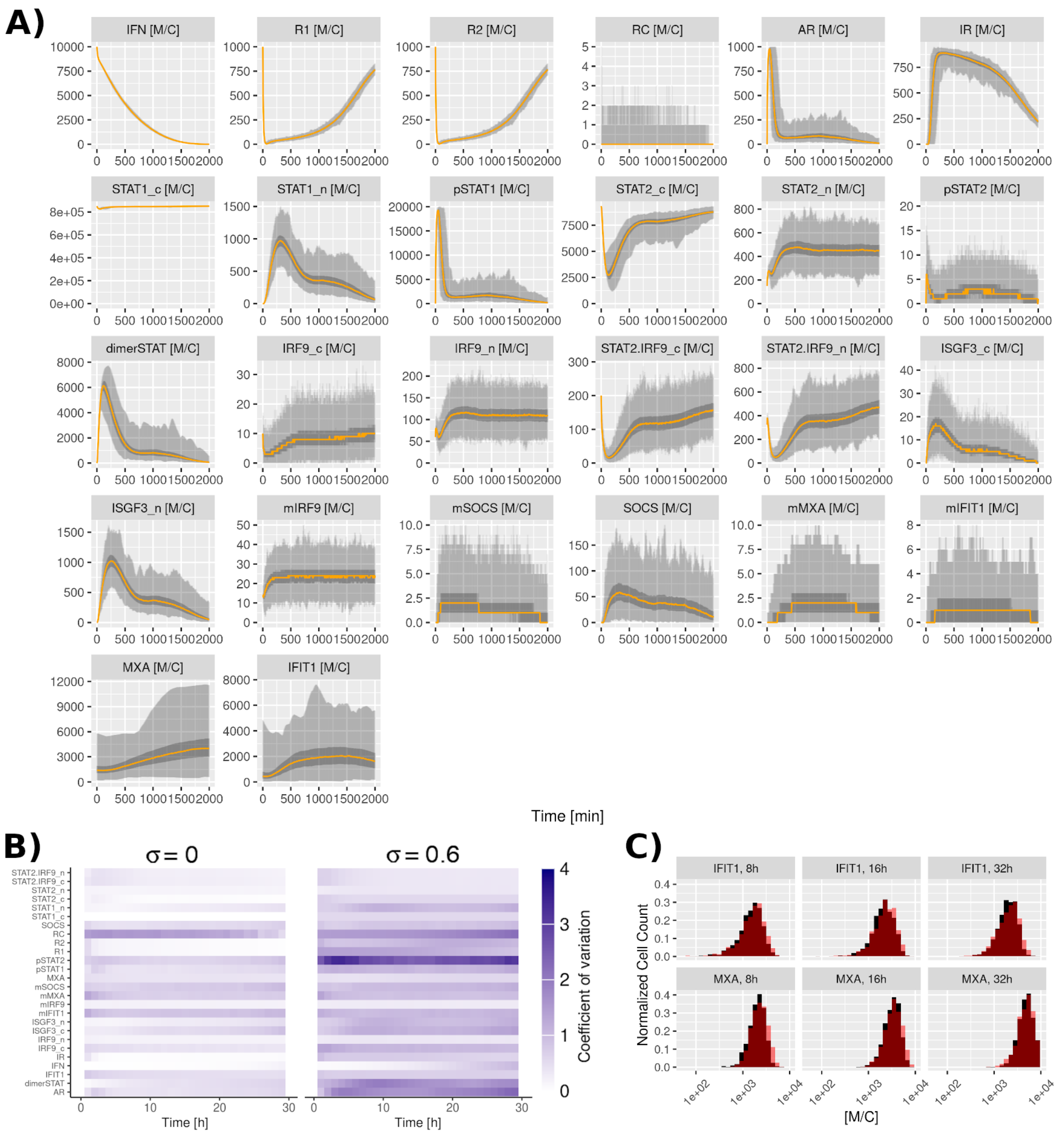

Figure O. Stochastic dynamics of cells with a nuclear-to-cytoplasmatic ratio of 27% (cf. 13.5% for healthy hepatocytes).
A) Model temporal stochastic dynamics for a system without extrinsic noise. Time course data describing the temporal dynamics of all species involved in the JAK-STAT signaling pathway. The plots represent the median values calculated with the repetitions of the stochastic model (orange lines). The y-axis has units of Molecules per Cell (M/C). The range of the distributions is indicated by the light gray ribbons, while dark gray ribbons represent 50% KI intervals. **B)** Variability in the JAK-STAT signaling pathway was measured during different time points and for all the elements that form the pathway. The effects of extrinsic noise in the system were calculated by the coefficient of variation ($cv = \sigma_S/(\mu_S + 0.1)$, where the subindex s represents the species in the pathway). In the plot the colorbar varies between 0 (white color) and larger than 4 (blue color), dark colors represent high variability in the dynamics of the studied species. The plots are consistent with those where a N:C ratio of 13.5% was assumed (Fig 5B). **C)** Stochastic simulations of different time points after IFN stimulation displaying a correct agreement in shape and location for multiple nuclear-cytoplasmic ratios. Simulated time-dependent distributions were computed by solving our model under stochastic dynamics using a distribution of values as initial conditions and repeating the simulations 1,000 times assuming a N:C ratio of 13.5% (black histogram) and 27% (red histogram), respectively. The red histogram is scaled by 24% to compensate for shifted steady state conditions.

H Abbreviations

AR, active Interferon receptor; a.u., arbitrary units; BAC, bacterial artificial chromosome; CDF, cumulative density function; dimerSTAT, heterodimer made by phosphorylated forms of STAT1 and STAT2; FACS, fluorescence activated flow cytometry; IFIT1, Interferon-induced protein with tetratricopeptide repeats 1; IFN, interferon; IR, inactive interferon receptor; IRF9, Interferon regulatory factor 9; ISG, IFN-stimulated gene; ISGF3, Interferon-stimulated gene factor 3; GA, genetic algorithm; GFP, green fluorescent protein; JAK, Janus kinase; KS-distance, Kolmogorov-Smirnov distance; MxA, Interferon-induced GTP-binding protein MxA; CDF, cumulative density function; pSTAT, phospho-signal transducer and activator of transcription; R1, Interferon receptor subunit 1; R2, Interferon receptor subunit 2; RC, Interferon receptor complex; SOCS, Suppressor of cytokine signaling; STAT, signal transducer and activator of transcription; SSA, stochastic simulation algorithm; UI, International Units.

Supplementary References

1. Cao Y, Gillespie DT, Petzold LR. Adaptive explicit-implicit tau-leaping method with automatic tau selection. Journal of Chemical Physics. 2007;126(22). doi:10.1063/1.2745299.

2. Hoops S, Sahle S, Gauges R, Lee C, Pahle J, Simus N, et al. COPASI–a COmplex PAthway SImulator. Bioinformatics (Oxford, England). 2006;22(24):3067–74. doi:10.1093/bioinformatics/btl485.

3. Gillespie DT. The chemical Langevin equation. The Journal of Chemical Physics. 2000;113(1):297–306.

4. Schnoerr D, Sanguinetti G, Grima R. Comparison of different moment-closure approximations for stochastic chemical kinetics. The Journal of chemical physics. 2015;143(18):11B610_1.

5. Maiwald T, Schneider A, Busch H, Sahle S, Gretz N, Weiss TS, et al. Combining theoretical analysis and experimental data generation reveals IRF9 as a crucial factor for accelerating interferon α-induced early antiviral signalling. FEBS journal. 2010;277(22):4741–4754.

6. Grandvaux N, Servant MJ, tenOever B, Sen GC, Balachandran S, Barber GN, et al. Transcriptional profiling of interferon regulatory factor 3 target genes: direct involvement in the regulation of interferon-stimulated genes. Journal of virology. 2002;76(11). doi:10.1128/jvi.76.11.5532-5539.2002.

7. Ashley CL, Abendroth A, McSharry BP, Slobedman B. Interferon-Independent Upregulation of Interferon-Stimulated Genes during Human Cytomegalovirus Infection is Dependent on IRF3 Expression. Viruses. 2019;11(3). doi:10.3390/v11030246.

8. Holzinger D, Jorns C, Stertz S, Boisson-Dupuis S, Thimme R, Weidmann M, et al. Induction of MxA Gene Expression by Influenza A Virus Requires Type I or Type III Interferon Signaling. Journal of Virology. 2007;81(14):7776–7785. doi:10.1128/JVI.00546-06.

9. Lambers WM, de Leeuw K, Doornbos-van der Meer B, Diercks GFH, Bootsma H, Westra J. Interferon score is increased in incomplete systemic lupus erythematosus and correlates with myxovirus-resistance protein A in blood and skin. Arthritis Res Ther. 2019;21. doi:10.1186/s13075-019-2034-4.

10. Maria NI, Brkic Z, Waris M, van Helden-Meeuwsen CG, Heezen K, van de Merwe JP, et al. MxA as a clinically applicable biomarker for identifying systemic interferon type I in primary Sjögren's syndrome. Annals of the Rheumatic Diseases. 2014;73(6):1052–1059. doi:10.1136/annrheumdis-2012-202552.

11. Lillacci G, Khammash M. The signal within the noise: efficient inference of stochastic gene regulation models using fluorescence histograms and stochastic simulations. Bioinformatics. 2013;29(18):2311–2319.

12. Aguilera LU, Zimmer C, Ursula K. A New Efficient Approach to Fit Stochastic Models on the Basis of High-throughput Experimental Data Using a Model of IRF7 Gene Expression as Case Study. BMC Systems Biology. 2017;11(26).

13. Lillacci G, Khammash M. Model selection in stochastic chemical reaction networks using flow cytometry data. In: 2011 50th IEEE Conference on Decision and Control and European Control Conference. IEEE; 2011. p. 1680–1685.

14. Scrucca L. GA: A Package for Genetic Algorithms in R. Journal of Statistical Software. 2013;53(4):1–37. doi:10.18637/jss.v053.i04.

15. Scrucca L. On Some Extensions to GA Package: Hybrid Optimisation, Parallelisation and Islands Evolution. The R Journal. 2017; p. 187–206. doi:10.32614/RJ-2017-008.

16. Ronni T, Matikainen S, Lehtonen A, Palvimo J, Dellis J, Van Eylen F, et al. The proximal interferon-stimulated response elements are essential for interferon responsiveness: a promoter analysis of the antiviral MxA gene. Journal of interferon & cytokine research. 1998;18(9):773–781.

17. Nakade K, Handa H, Nagata K. Promoter structure of the MxA gene that confers resistance to influenza virus. FEBS letters. 1997;418(3):315–318.

18. Begitt A, Droescher M, Meyer T, Schmid CD, Baker M, Antunes F, et al. STAT1-cooperative DNA binding distinguishes type 1 from type 2 interferon signaling. Nature immunology. 2014;15(2):168–76. doi:10.1038/ni.2794.

19. Rand U, Rinas M, Schwerk J, Nöhren G, Linnes M, Kröger A, et al. Multi-layered stochasticity and paracrine signal propagation shape the type-I interferon response. Molecular systems biology. 2012;8(1):584.
